# Inflammatory responses relate to distinct bronchoalveolar lavage lipidome in community-acquired pneumonia patients: a pilot study

**DOI:** 10.1186/s12931-019-1028-8

**Published:** 2019-05-02

**Authors:** Yali Zheng, Pu Ning, Qiongzhen Luo, Yukun He, Xu Yu, Xiaohui Liu, Yusheng Chen, Xiaorong Wang, Yu Kang, Zhancheng Gao

**Affiliations:** 1 Department of Pulmonary and Critical Care, Peking University People’s Hospital, Beijing, China; 2 Department of Pulmonary and Critical Care, the Second Affiliated Hospital of Xi’an Jiaotong University, Xi’an, China; 3 National Protein Science Technology Center, Tsinghua University, Beijing, China; 4 Department of Pulmonary and Critical Care, Fujian Provincial Hospital, Fuzhou, China; 5 Department of Pulmonary and Critical Care, Union Hospital, Tongji Medical College, Huazhong University of Science and Technology, Wuhan, China; 6 CAS Key Laboratory of Genome Sciences and Information, Beijing Institute of Genomics, Chinese Academy of Sciences, Beijing, China

**Keywords:** Community-acquired pneumonia, Lipidomic profile, Bronchoalveolar lavage, Bioactive lipid, Inflammatory response, Phagocyte

## Abstract

**Background:**

Community-acquired pneumonia (CAP) is a leading cause of morbidity and mortality worldwide. Antibiotics are losing their effectiveness due to the emerging infectious diseases, the scarcity of novel antibiotics, and the contributions of antibiotic misuse and overuse to resistance. Characterization of the lipidomic response to pneumonia and exploring the “lipidomic phenotype” can provide new insight into the underlying mechanisms of pathogenesis and potential avenues for diagnostic and therapeutic treatments.

**Methods:**

Lipid profiles of bronchoalveolar lavage fluid (BALF) samples were generated through untargeted lipidomic profiling analysis using high-performance liquid chromatography with mass spectrometry (HPLC-MS). Principal component analysis (PCA) was applied to identify possible sources of variations among samples. Partitioning clustering analysis (*k*-means) was employed to evaluate the existence of distinct lipidomic clusters.

**Results:**

PCA showed that BALF lipidomes differed significantly between CAP (*n* = 52) and controls (*n* = 68, including 35 healthy volunteers and 33 patients with non-infectious lung diseases); while no clear separation was found between severe CAP and non-severe CAP cases. Lactosylceramides were the most prominently elevated lipid constituent in CAP. Clustering analysis revealed three separate lipid profiles; subjects in each cluster exhibited significant differences in disease severity, incidence of hypoxemia, percentages of phagocytes in BALF, and serum concentrations of albumin and total cholesterol (all *p* < 0.05). In addition, SM (d34:1) was negatively related to macrophage (adjusted r = − 0.462, *p* < 0.0001) and PE (18:1p/20:4) was positively correlated with polymorphonuclear neutrophil (PMN) percentages of BALF (adjusted r = 0.541, *p* < 0.0001). The 30-day mortality did not differ amongst three clusters (*p* < 0.05).

**Conclusions:**

Our data suggest that specific lower airway lipid composition is related to different intensities of host inflammatory responses, and may contribute to functionally relevant shifts in disease pathogenesis in CAP individuals. These findings argue for the need to tailor therapy based on specific lipid profiles and related inflammatory status.

**Trial registration:**

ClinicalTrials.gov (NCT03093220). Registered on 28 March 2017 (retrospectively registered).

**Electronic supplementary material:**

The online version of this article (10.1186/s12931-019-1028-8) contains supplementary material, which is available to authorized users.

## Introduction

Community-acquired pneumonia (CAP), an acute infection of the pulmonary parenchyma acquired outside of a health care setting, is a leading cause of morbidity and mortality worldwide, especially among geriatric populations [1–3]. The incidence of CAP and risk of death are linked to increasing age and the presence of comorbidities [2, 4]. Despite the availability of effective antibiotics and improved sophisticated diagnostic techniques, CAP remains a big challenge in the era of global aging [5].

Inflammation is an essential defense part of the body’s response to infection. It helps clear the invading microorganisms but can also induce host tissue damage and disease. Thus, an effective but not excessive inflammatory response may help to improve the outcome of CAP. Bioactive lipids are known to play crucial roles in the pathophysiology of inflammation, changes in their concentrations affect cell functions, often intracellular trafficking and signaling, cell adhesion, migration, and apoptosis [6]. Moreover, novel lipid mediators derived from polyunsaturated fatty acids (PUFAs), including lipoxins, resolvins, protectins, and maresins, have drawn great attention for their dual anti-inflammatory and pro-resolving effects in inflammation [7, 8]. Bioactive lipids and their derivatives could be promising therapeutic targets for CAP in the near future [9].

In a previous study, we described the serum metabolic profiles of CAP and found that the sphingolipid metabolism pathway was significantly dysregulated in pneumonia [10]. Furthermore, the serum level of sphinganine was significantly elevated in severe CAP (SCAP), indicating a potential key role for this sphingolipid in the pathogenesis of pneumonia. In order to characterize the changes in these bioactive lipids during pneumonia, we applied untargeted lipidomic profiling on bronchoalveolar lavage fluid (BALF) samples. Given that BALF has been described as a “liquid biopsy” for its diagnostic utility, this noninvasive approach represented the most effective means to uncover a lung-specific lipidomic response in vivo. We conducted principal component analysis (PCA) to identify correlations between BALF lipidomes and clinical features of patients, as well as clustering analysis to explore the existence of lipid clusters. We found specific correlations between levels of two lipid species and prevalent phagocytes in BALF, as well as three distinct lipid profiles that correlate with degree of airway inflammation. This pilot study introduces the possible utility of BALF lipidome profiles as a future aid in tailoring of CAP therapies.

## Materials and methods

### Study populations

**Fig. 1:**
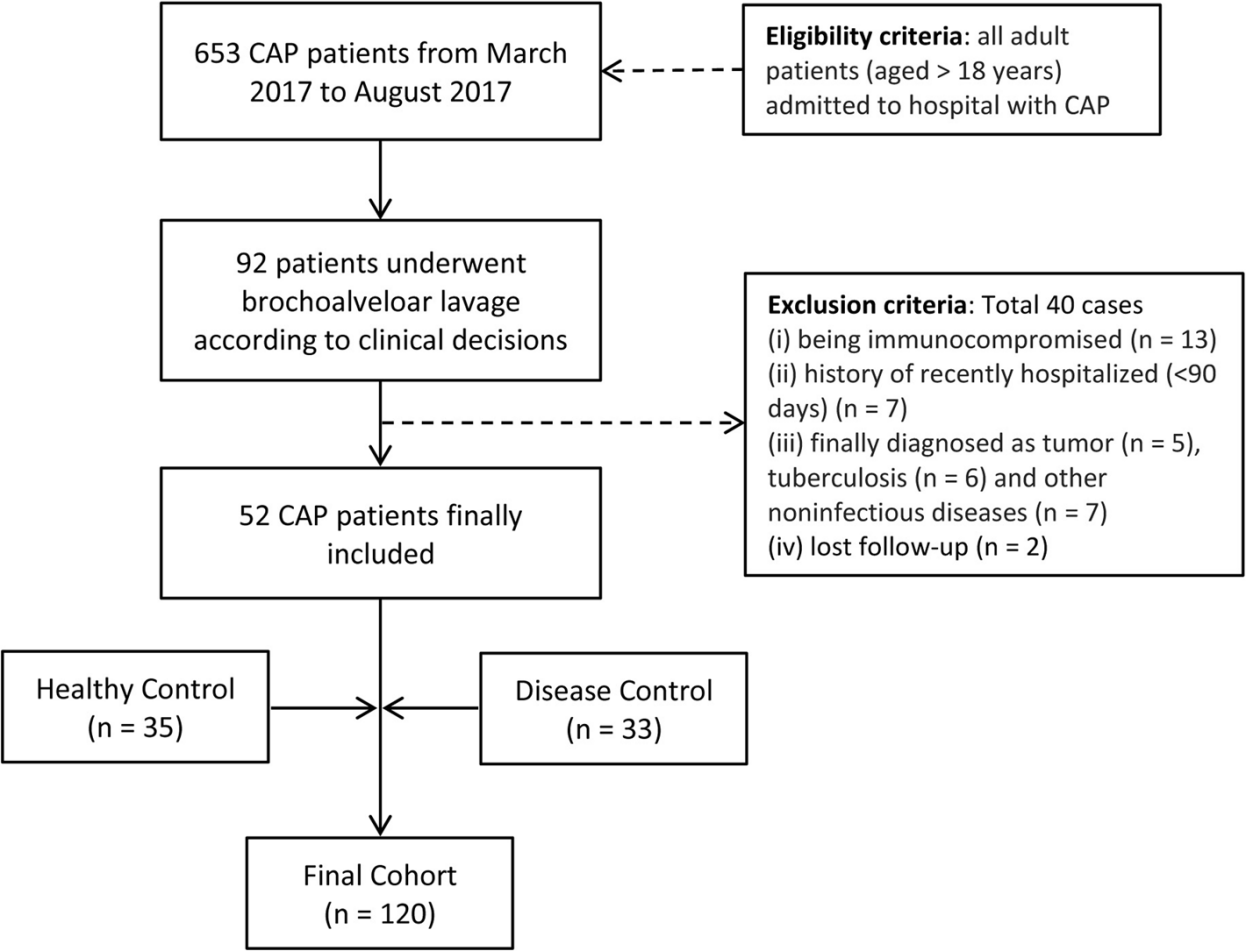
Flowchart of study enrollment

We enrolled 52 CAP patients admitted to any of the four participating hospitals between March 2017 and August 2017 as part of a multicenter clinical study (ClinicalTrials.gov, NCT03093220). During this period, we recruited thirty-five healthy volunteers and thirty-three patients with non-infectious pulmonary involvement such as connective tissue disease-associated interstitial disease (CTD-ILD) as disease controls. CAP and SCAP were defined according to the standard published by America/American Thoracic Society in 2007 [11]. Criteria for inclusion and exclusion are detailed in Fig. 1. General participant demographics, including age, gender, complications, laboratory findings, and clinical treatments, were collected using a standard form. The primary outcome was mortality follow-up at 30-days post-bronchoscopy. Individuals who aged over 65 were defined as the elderly. The ethical committee of Peking University People’s Hospital approved the research. All subjects provided written informed consent prior to the collection of any data.

### BALF sample collection and preservation

Bronchoscopy was performed within 72 h after hospital admission. Clinicians determined the necessity and feasibility of bronchoscopy individually. Briefly, two 50 mL aliquots (100 mL total) of sterile normal saline were instilled into the diseased region according to CT scan results (for CAP patients), or into the right middle lobe or lingual (for controls). Aliquots were then retrieved by gentle suctioning through the suction port of the bronchoscope. No suction was performed before sample collection. Total and differential cell counts, etiological examination, and protein concentration of BALF samples were analyzed respectively at the time of collection in local labs. Protein in the BALF samples was measured with the bicinchoninic acid (BCA) assay (Pierce, Thermo Fisher Scientific, Rockford, IL USA). The rest BALF samples were clarified by centrifugation at 10,000×g for 10 min at 4 °C and the supernatants and precipitates were separated and frozen (− 80 °C) until the time of assay.

### Untargeted lipidomic profiling analysis based on HPLC-MS

#### BALF sample preprocessing

BALF samples were thawed on ice at the time of assay. The sample volume used in high-performance liquid chromatography-mass spectrometry (HPLC-MS) analysis was normalized by its protein concentration to get an equal mass for all samples [12]. The equation was as follows: Sample volume (μL) = a constant mass (g)/ BALF protein concentration (g/L) × 1000. A methyl tert-butyl ether (MTBE)-based extraction protocol [13] was used. For deproteinization, samples were freeze-dried (CentriVap®) and then combined with 200 μL of pre-chilled 75% methanol containing 0.5 μg/mL L-Tryptophan-(*indole*-d5) which served as an internal control. The sample was combined with 500 μL of MTBE and vortexed. Then the mixtures were incubated for 1 h at room temperature. 125 μL of HPLC-grade water was added, then centrifuged (15 min at 14,000×g, 4 °C). The upper non-polar fraction was transferred to a new 1.8 mL Eppendorf tube, then freeze-dried and stored at − 80 °C until untargeted LC-MS analysis. Quality control (QC) samples were prepared by pooling 20 μL aliquots from each sample and extracted as above.

#### Untargeted HPLC-MS analysis and compounds identification

HPLC-MS analysis was performed using a Cortecs C18 column (2.1 × 100 mm, Waters) on an Ultimate 3000 UHPLC (Dionex) system coupled with Q Exactive (Orbitrap) mass spectrometer (Thermo Fisher, CA). The dried samples were reconstituted in chloroform:methanol (2:1 *v*/v) and transferred to an autosampler vial for analysis. Before experimental sample analyses, six QC samples were injected to stabilize the instrument. All samples were processed in random order and were assigned to an HPLC-MS run in random order using a computerized algorithm, with a QC sample between every 10 experimental samples. Detailed parameters for the untargeted lipid analyses were set following the protocols of our previously reported study [14] and described in the Additional file 1: Supplementary Method. Data-dependent MS/MS acquisition (DDA) of all samples was analyzed using TraceFinderTM (Thermo, CA). Lipids were assigned using an in-house lipid database in “screening” mode and qualified in “quan” mode. Lipids were identified based on matching precursor and characteristic fragment masses. Five ppm and 10 ppm mass tolerance was used for precursor and fragment, respectively. Only the lipids with chromatographic area > 5 × 10^6^ were considered as confident identification. A 0.25 min retention time shift was allowed for quantitation.

### HPLC-MS data analysis workflow

#### Data preprocessing

First, a lipid was kept if it had a non-zero value for at least 80% in the samples of any one group (CAP or controls) [15, 16]. Second, missing values were replaced by a small value (half of the minimum positive value in the original data), assuming that most missing values are caused by low abundance compounds (i.e., below the detection limit) [16, 17]. Afterward, to reduce the large differences between the total amounts of compounds among the diverse samples and to increase the contribution of lower concentration metabolites in the generated models, data were normalized to the constant sum and then auto scaled [18] (mean-centered and divided by the standard deviation of each variable) using MetaboAnalyst 4.0 [19]. The normalized data were used for downstream analysis.

### Data analysis

Unsupervised principal component analysis (PCA) was performed using SIMCA-P software version 14.0 (Umetrics, Umea, Sweden) in order to find distribution trends and possible sources of variation among samples. Subsequently, to evaluate if the host-lipidome in BALF could be partitioned into clusters with distinct lipidomic phenotypes, *k-*means partitional clustering [20] was employed based on the lipid profiles of all subjects using MetaboAnalyst 4.0. An optimal number of clusters *k* is the one that maximizes the average silhouette over a range of possible values for *k*. The elbow method [21] was used to determine the optimal number of clusters using the R package “NbClust” [22]. The idea of the elbow method is to run *k*-means clustering on the dataset for a range of values of *k*, and for each value of *k* calculate the sum of squared errors (SSE). For correlation studies, the two-tailed Spearman test was used to calculate correlation significance. Multiple linear regression (MLR) analysis was then conducted using a stepwise method to adjust multi-colinearity inherent in lipidomic data.

### Statistical analysis

All categorical variables are presented as numbers (percentages), parametric continuous variables are presented as mean ± SD, and nonparametric continuous variables are presented as median and interquartile ranges (25th and 75th percentiles). Student’s t-test or analysis of variance (ANOVA) with post-hoc Tukey HSD test were used to analyze continuous parametric data, whereas continuous nonparametric data were analyzed using Mann-Whitney U or a Kruskal-Wallis test. All categorical data were analyzed using chi-square or Fisher’s exact test. Analyses were performed in SPSS Statistics (version 22.0) or MetaboAnalyst 4.0 [19]. A nominal *p*-value of less than 0.05 was considered to be of statistical significance for clinical data. Benjamini-Hochberg (BH) adjusted p-value of less than 0.05 was defined as significant for HPLC-MS data.

## Results

### Lipid profiles of human BALF

BALF lipid profiles of 120 subjects were generated through untargeted lipidomic profiling analysis using HPLC-MS. The final study population consisted of 52 patients with CAP, 35 healthy individuals and 33 patients with connective tissue disease-associated interstitial lung disease (CTD-ILD) as disease controls (Fig. 1). As indicated in Table 1, there was a range of etiologies of CAP including bacteria, virus, and fungus, which was typical for a heterogeneous CAP patient population. Additional demographic data about these patients are detailed in Table 1.

**Table 1:** Demographical and Clinical features of included subjects

	SCAP (*n* = 21)	NSCAP (*n* = 31)	Controls (*n* = 68)	*p*-value
Age	58.48 ± 17.37	49.53 ± 23.07	53.43 ± 12.45	0.164
Gender, n, (% male)	16 (76.2%)	17 (54.8%)	20 (29.4%)	<0.0005^a,b^
Ever smokers, n (%)	4 (19.0%)	4 (12.9%)	2 (2.9%)	0.019
Current Smokers, n (%)	6 (28.6%)	5 (16.1%)	5 (7.4%)	0.035
Comorbidities				
Diabetes Mellitus, n (%)	5 (23.8%)	3 (9.7%)	11 (16.2%)	0.398
Hypertension, n (%)	6 (28.6%)	10 (32.3%)	21 (30.9%)	1.0
Hyperlipidemia, n (%)	2 (9.5%)	9 (29.0%)	8 (11.8%)	0.089
Coronary Heart Disease, n (%)	4 (19.0%)	3 (9.7%)	8 (11.8%)	0.643
Laboratory findings of BALF				
Total cell counts (× 10^6^ cells)	0.2 (0.18 - 0.2)	0.2 (0.18 - 0.35)	0.2 (0.13 - 0.29)	0.704
PMN percentages (%)	41.0 (2.0 - 65.0)	4.5 (1.5 - 28.0)	1.5 (1.0 - 4.5)	<0.0005^a,b^
Macrophage percentages (%)	35.0 (14.0 - 58.0)	50.0 (32.0 - 70.5)	72.25 (43.5 - 88.0)	<0.0005^a,b^
Lymphocyte percentages (%)	20.0 (13.0 - 27.0)	19.75 (10.0 - 42.0)	21.5 (9.25 - 41.0)	0.948
Eosinophil percentages (%)	0 (0 - 0)	0 (0 - 1)	0 (0 - 0.75)	0.320
Albumen concentration (g/L)	1.45 (0.89-2.36)	0.43 (0.10 - 1.92)	0.17 (0.08 – 0.27)	<0.0005^a,b,c^
Detected pathogen				
Bacteria	5 (23.8%)	0 (0)	NA	0.017
Atypical pathogen	5 (23.8%)	9 (29.0%)	NA	0.677
Virus	6 (28.6%)	11 (35.5%)	NA	0.602
Fungus	2 (9.5%)	2(6.5%)	NA	0.903
Unknown	7 (33.3%)	15 (48.4%)	NA	0.281

Abbreviations: *BALF* bronchoalvolar lavage, *PMN* polymorphonuclear leukocyte, *NA* not available. Data are presented as n (%) for categorical data, mean (±SD) for parametrically distributed data, or median (interquartile range) for nonparametrically distributed data. Statistically significant differences in variables amongst three groups were calculated using one-way ANOVA with post-hoc Turkey HSD test or Kruskal-Wallis H test for continuous data; and Fisher’s exact test with a Bonferroni correction for categorical data. ^a^ statistically significance exists between SCAP and Control; ^b^ statistically significance exists between NSCAP and Control; ^c^ statistically significance exists between SCAP and NSCAP

**Fig. 2:**
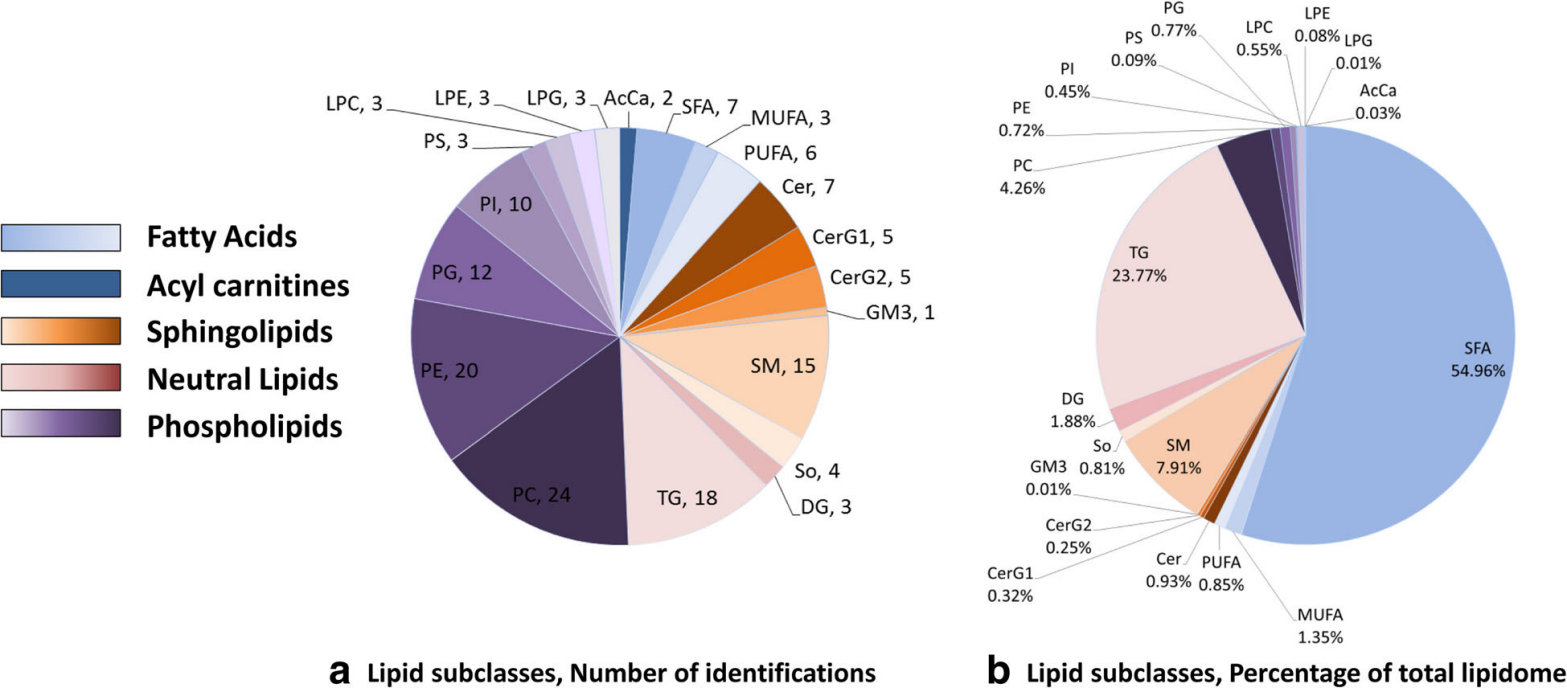
Overview of bronchoalveolar lavage fluid (BALF) lipidome. **a** A total of 150 lipid species are detected in BALF samples of community-acquired pneumonia (CAP) and Controls. The lipids are categorized into 5 lipid classes: fatty acids (light blue), acylcarnitines (dark blue), sphingolipids (orange), neutral lipids (pink), and phospholipids (purple). **b** The compositions of total BALF lipid signal. Proportions of each lipid subclass are calculated by normalizing to total lipid intensities

Overall, mass spectrometry detected 150 lipid species in these samples, 65 in positive and 108 in negative electrospray ionization (ESI) mode. The lipids were categorized into 5 lipid classes: fatty acids (FA), neutral lipids, sphingolipids, phospholipids and acylcarnitines (Fig. 2a, Additional file 2: Table S1). Acylcarnitines, sphingolipid, and neural Lipid were more effectively detected in positive ion mode; while FA and phospholipids were more effectively detected in negative ion mode. In BALF samples, FAs contributed 57.15% to the total lipid signal; followed by neural lipids (25.65%), sphingolipids (10.23%), and phospholipids (6.94%). The top 4 dominant lipid subclasses comprised over 90% of the total lipid signal (Fig. 2b, Additional file 2: Table S1), including saturated FA (SFA, 54.96%), triglyceride (TG, 23.77%), sphingomyelin (SM, 7.91%), and phosphatidylcholine (PC, 4.26%).

### Lipidomic alterations in CAP

Kruskal-Wallis test identified ten lipid subclasses (Table 2) that were significantly different in SCAP patients, compared to controls (adjusted *p*-value < 0.05). Significantly increased BALF level of lactosylceramide (CerG2, > 10-fold in relative abundance), monounsaturated FA (MUFA, > 2-fold), polyunsaturated FA (PUFA, > 2-fold), phosphatidylethanolamine (PE, > 2-fold), and diglyceride (DG, ~ 2-fold) were observed in SCAP patients, while sphingosine (So), phosphatidylglycerol (PG), lysophosphatidylcholine (LPC), lysophosphatidylethanolamine (LPE), and lysophosphatidylglycerol (LPG) were notably decreased ~ 0.5-fold. No significantly differences in lipid subclasses were observed between the SCAP and the NSCAP groups.

**Table 2:** Comparisons of lipid subclasses between CAP and controls

Lipid Class	Lipid Sub-Class	Adduct	% of the total lipid signals	Fold change			Kruskal-Wallis adjusted *p*-value
				SCAP/Control	NSCAP/ Control	SCAP/NSCAP	
Acylcarnitines	AcCa	+H	0.06%	0.48	0.75	0.63	0.078
Fatty acids	SFA	-H	54.96%	0.93	0.99	0.94	0.14
	MUFA	-H	1.35%	2.13^***^	1.13^**^	1.92	< 0.0005
	PUFA	-H	0.85%	2.50^*^	1.33^*^	1.92	0.004
Sphingolipids	Cer	+H	2.16%	1.09	1.41	0.81	0.95
	CerG1	+H	0.32%	1.13	1.46	0.82	0.482
	CerG2	+H	0.25%	16.36^***^	13.51^***^	1.64	< 0.0005
	GM3	+H	0.01%	0.98	1.16	0.88	0.125
	SM	+H	7.91%	1.11	1.06	1.06	0.792
	So	+H	0.81%	0.44^***^	0.65^**^	0.67	< 0.0005
Neutral lipids	DG	+NH4	1.88%	1.96^*^	1.96	0.94	0.031
	TG	+NH4	23.77%	0.92	0.91	1.00	0.222
Phospholipids	PC	+CH3COO	4.26%	1.36	1.01	1.36	0.81
	PE	-H	0.72%	2.14^***^	1.43^*^	1.60	< 0.0005
	PG	-H	0.77%	0.67^*^	0.87	0.76	0.019
	PI	-H	0.45%	1.65	0.90	1.84	0.110
	PS	-H	0.09%	1.69	1.50	1.20	0.034
	LPC	+H	0.55%	0.47^**^	0.71	0.70	0.002
	LPE	+H	0.08%	0.49^*^	0.76	0.70	0.014
	LPG	-H	0.01%	0.41^***^	0.72	0.58	< 0.0005

The relative abundances of lipid subclasses were calculated from the sum of lipid species that classified to the same subclass. The * depicts a statistically significant difference. * *p* < 0.05; ** *p* < 0.01; *** *p* < 0.001

**Fig. 3:**
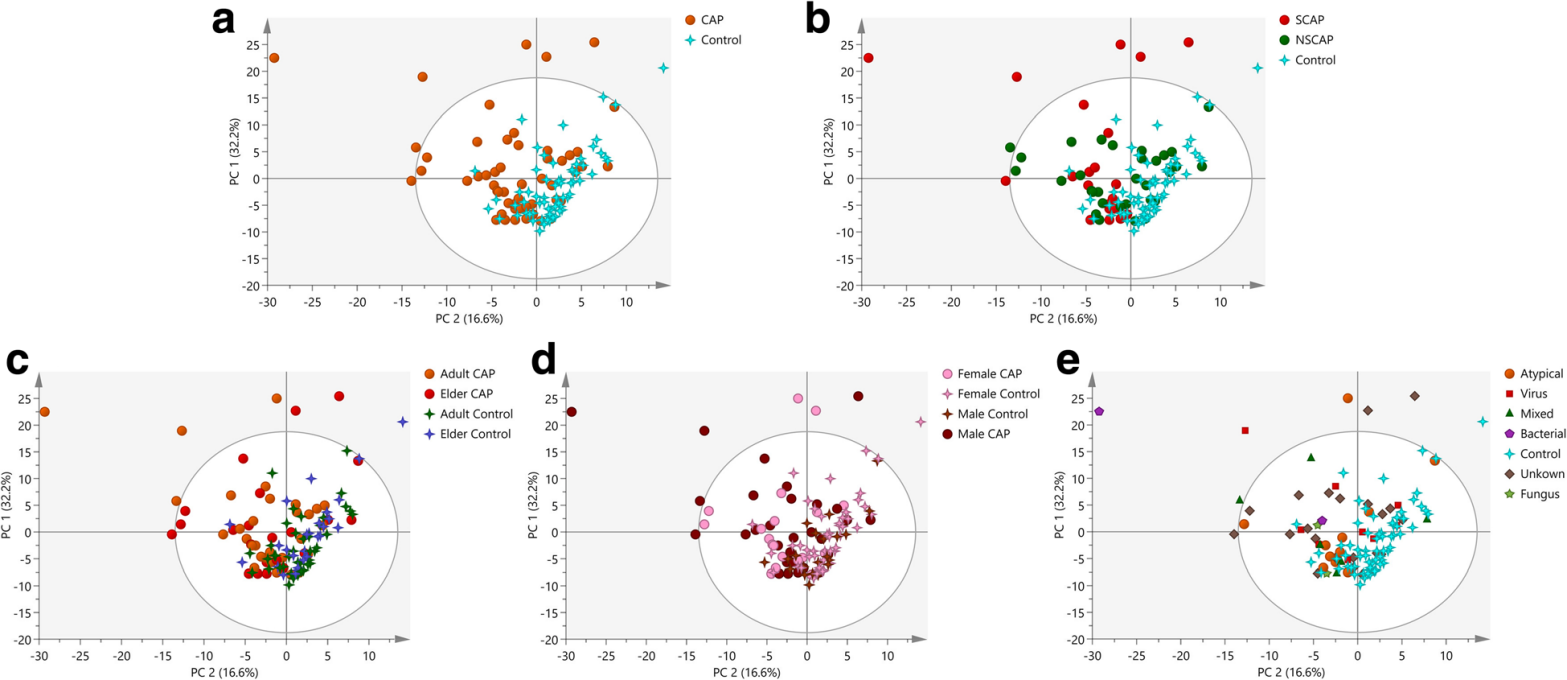
Lipid profiles of CAP patients and controls. **a** Principal component analysis (PCA) scores plot of lipidomic profiles in BALF samples. PCA scores plot colored according to sample group: red circles, severe CAP (SCAP); green circles, non-severe CAP (NSCAP); turquoise four-point stars, healthy control (HC); and yellow triangles, quality control (QC) samples. The PCA model (R2X = 0.871, Q2 = 0.76) reflect good separation trends among SCAP and controls. Classifications based on disease severity (SCAP vs NSCAP, **b**), age (adult CAP vs elder CAP, **c**), gender (male CAP vs female CAP, **d**), and causative pathogens (viral, bacterial, fungal, or mixed infection, **e**) revealed indistinct separation trends, suggesting that the major clinical-demographic features are not the sole defining features of these BALF lipids

All identified lipids were subjected to PCA in SIMCA 14.1 to explore the major effects that potentially drive the differences in lipid profiles in CAP patients. The PCA resulted in a ten-component model, R2X _(cum)_ = 0.871 and Q2 _(cum)_ = 0.76. As shown in Fig. 3a, samples in the control group clustered tightly, while samples in the CAP group were distributed diffusely, thus revealing high heterogeneity in the lipids that accompany this disease. Classifications based on disease severity (SCAP vs NSCAP, Fig. 3b), age (adult CAP vs elder CAP, Fig. 3c), gender (male CAP vs female CAP, Fig. 3d), and causative pathogens (viral, bacterial, fungal, or mixed infection, Fig. 3e) revealed indistinct separation trends, suggesting that individually, the major clinical-demographic features do not contribute strong effects to the clustering patterns of these BALF lipids.

Thirty-three lipid species were identified (32 higher and 1 lower, Additional file 2: Table S2) that displayed differential relative abundance amongst SCAP, NSCAP, and Control (ANOVA with Turkey host HSD test, FDR < 0.05), consisting of 3 sphingolipid, 6 FA, and 24 phospholipid species. Overall, most of the significantly different lipids were elevated in SCAP subjects (including SM, FA, phosphatidylcholine (PC), PE, and phosphatidylinositol (PI) species). Only palmitic acid (FA 16:0), which comprised 25.96% of the total lipid signal, was significantly decreased in SCAP. Few lipid species were differentially expressed between SCAP and NSCAP, including SM (d34:1), PE (16:0p/20:4), and PC (18:0/20:3). Subsequently, to determine the predictive ability of these differential lipids as biomarkers for SCAP, we applied receiver operating characteristic (ROC) analysis and compared their areas under the curve (AUC). As shown in Additional file 3: Figure S1, FA (24:0), FA (20:1), FA (18:1), and FA (16:1) exhibited good performances in discriminating SCAP from NSCAP patients (AUC = 0.747, AUC = 0.737, AUC = 0.757, and AUC = 0.747, respectively).

### Unsupervised clustering revealed three distinct lipidomic clusters (LCluster) correlated with different inflammatory responses and disease severity

**Fig. 4:**
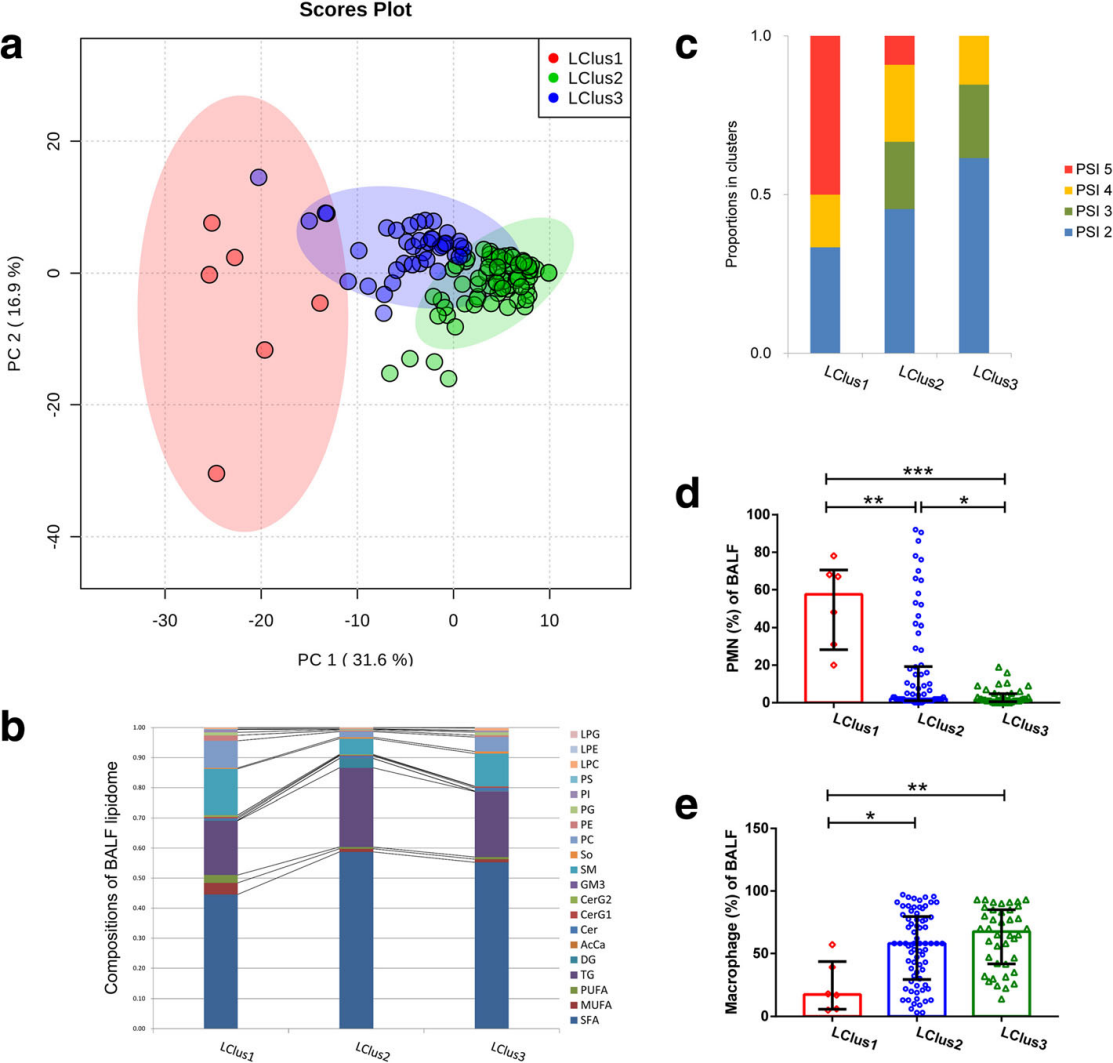
Panel **a**: PCA scores plot of three distinct lipid clusters (LCluster). Red, LClus1; blue, LClus2; green, LClus3. Panel **b**: Distinct compositions of lipid subclasses amongst three clusters. Panel **c**: The distributions of CAP patients with different PSI classes amongst three clusters. PSI 2 (blue section), 51–70 points; PSI 3 (green section), 71–90 points; PSI 4 (yellow section), 91–130 points; and PSI 5 (red section), 131–395 points. Panel **d**-**e**: Comparisons of macrophage percentages (**d**) and PMN percentages of BALF (**e**) amongst the three clusters. LClus1 exhibits the highest percentage of polymorphonuclear leukocytes (PMNs) and the lowest percentage of macrophages in BALF

To evaluate whether BALF samples from all cohorts could be partitioned into clusters with distinct lipidomic phenotypes, we performed the *k-means* algorithm and the *k* number was determined by the elbow method (Additional file 3: Figure S2). This approach yielded three separate lipid clusters (LClusters) (Fig. 4a). Clinical and laboratory characteristics were compared in detail amongst the three clusters (Table 3): LClus1, LClus2, and LClus3. The percentages of polymorphonuclear leukocytes (PMN) in BALF significantly differed among the three clusters, with the highest value in LClus1 and the lowest in LClus3 (Fisher’s exact test, *p* < 0.0001, Fig. 4d); while the percentages of macrophage revealed an opposite tendency (Fig. 4e). Since PMN infiltration plays a central role in inflammation and is a major cause of tissue damage, we stratified our subjects into high- (LClus1), medium- (LClus2), and low- (LClus3) inflammatory response groups. Patients in LClus1 were characterized by lower serum concentrations of albumin (ALB) and total cholesterol (TC) (Fisher’s exact test, *p* < 0.01). The incidence of hypoxemia was significantly higher in LClus1 (6/6, 100%) than in the other two groups (17.57% in LClus2 and 7.5% in LClus3, *p* = 0.008 and 0.005, respectively). Half of the patients in LClus1 received invasive positive pressure ventilation (IPPV) therapy (Fisher’s exact test, *p* = 0.022), which is the highest ratio of three clusters, although without statistical significance in the following pairwise comparisons. In addition, patients in LClus1 were predicted to have the highest mortality (defined as Pneumonia Severity Index (PSI) class = 5, Fig. 4c). Based on the above findings, we presumed that the different intensities of inflammatory responses were also associated with diverse disease severity degrees. Goodman’s & Kruskal’s Gamma test showed statistically significant positive correlations between the inflammatory response rankings (high-, medium-, and low-) and the degree of disease severity, which were assessed using the following severity scoring systems: PSI (*p* = 0.045, G = 0.429), CURB-65 (*p* = 0.034, G = 0.446) and APACHE II (*p* = 0.007, G = 0.565), respectively. However, patients in the high- and the medium- response groups both revealed high mortality rate (16.7% vs 15.2%, Fisher’s exact test, *p* > 0.05). No difference was observed in the spectrum of pathogen amongst clusters.

**Table 3:** Demographical and clinical features of all subjects in three lipid clusters

	LClus1 (*n* = 6)	LClus2 (*n* = 74)	LClus3 (*n* = 40)	*p*-value
Cluster composition				
CAP patients, n (%)	6 (100%)	33 (44.6%)	13 (32.5%)	0.005^a,b^
SCAP patients, n (%)	6 (100%)	14 (18.9%)	1 (2.5%)	< 0.0005^a,b,c^
Demographics				
Age (years)	55.17 ± 16.13	53.49 ± 16.88	52.77 ± 16.78	0.935
Gender, n, (% male)	4 (66.7%)	35 (47.3%)	14 (35.0%)	0.243
Ever smokers, n (%)	1 (16.7%)	6 (8.1%)	3 (7.5%)	0.600
Current smokers, n (%)	1 (16.7%)	12 (16.2%)	3 (7.5%)	0.390
Underlying Diseases				
Diabetes Mellitus, n (%)	2 (33.3%)	12 (16.2%)	5 (12.5%)	0.376
Hypertension, n (%)	1 (16.7%)	25 (33.8%)	11 (27.5%)	0.661
Hyperlipidemia, n (%)	0	13 (17.6%)	6 (15.0%)	0.692
Coronary Heart Disease, n (%)	2 (33.3%)	9 (12.2%)	4 (10.0%)	0.281
Laboratory Findings				
Peripheral blood related				
BUN (mmol/L)	4.4 (1 - 27)	4.4 (1 - 25)	4.4 ± 1.5	0.323
Cr (μmol/L)	86.5 (30 - 448)	60 (30 - 237)	61.9 ± 18.4	0.345
ALT (U/L)	24.5 (12.0 - 77.5)	25.5 (13.3 - 44.5)	28.0 (18.0 - 41.0)	0.958
AST (U/L)	30.5 (14.8 - 248.5)	25 (18.0 - 32.0)	28.0 (21.0 - 43.0)	0.422
CK (U/L)	41.5 (19.0 - 540.0)	58.5 (43.3 - 94.8)	84 (48.0 - 116.0)	0.765
ALB (g/L)	25.4 ± 5.2	35.3 ± 6.7	35.8 ± 5.5	0.006^a,b^
Glucose (mmol/L)	9.4 ± 4.5	5.1 (4.7 - 6.2)	4.8 (4.5 - 5.7)	0.086
TC (mmol/L)	2.8 ± 1.0	3.8 (3.7 - 4.7)	4.8 ± 1.3	< 0.0005^a,b,c^
TG (mmol/L)	1.0 (0.7 - 2.8)	1.2 (0.9 – 1.6)	1.8 ± 1.0	0.166
WBC (× 10^9^/L)	11.3 (6.3 - 17.0)	6.1 (5.1 - 8.4)	6.7 ± 2.1	0.241
Neutrophils (× 10^9^/L)	8.9 ± 4.7	4.0 (3.1 - 6.0)	4.5 ± 1.7	0.105
Lymphocytes (× 10^9^/L)	1.0 ± 0.4	1.3 ± 0.6	1.5 ± 0.9	0.08
NLR	11.5 ± 9.7	2.9 (2.2 - 5.3)	3.9 ± 2.6	0.092
BALF related				
Total cell counts (× 10^6^ cells)	0.48 (0.18 – 0.74)	0.2 (0.16 -0.29 )	0.2 (0.14 - 0.25)	0.182
PMN percentages (%)	57.5 (28.3 – 70.5)	2 (1 - 19.3)	1.5 (0 - 4.0)	< 0.0005^a,b,c^
Macrophage percentages (%)	23.7 ± 20.4	54.7 ± 28.2	63.9 ± 24.0	0.006^a,b^
Lymphocyte percentages (%)	14.5 (9.8 – 38.0)	20 (9.0 – 37.0)	24 (13.0 – 48.0)	0.195
Eosinophil percentages (%)	0 (0 - 1)	0 (0 - 1)	0 (0 - 0.5)	0.979
Albumen concentration (g/L)	4.01 (0.87 – 6.05)	0.35 (0.17 – 1.16)	0.08 (0.06 – 0.22)	< 0.0005^a,b,c^
Inflammatory markers^d^				
PCT (μg/L)	17.9 ± 26.0	0.17 (0.05 - 0.37)	0.17 (0.11 - 0.21)	0.008^a,b^
CRP (mg/L)	83.7 ± 65.3	43.82 (13.8 - 117.0)	43.82 (11.7 - 88.4)	0.611
ESR (mm/h)	21 (18.3 - 41)	50 (23.5 - 16.5)	30.9 ± 17.6	0.209
Medication history				
Recent corticosteroid usage^e^	1 (16.7%)	5 (15.6%)	0	0.335
Recent antibiotic usage^e^	6 (100%)	31 (96.9%)	12 (92.3%)	0.611
Quinolones at bronchoscopy	4 (66.7%)	21 (65.6%)	6 (46.2%)	0.484
Macrolides at bronchoscopy	1 (16.7%)	3 (9.4%)	4 (30.8%)	0.206
β-lactams at bronchoscopy	1 (16.7%)	16 (50.0%)	6 (46.2%)	0.419
Carbapenems at bronchoscopy	4 (66.7%)	6 (18.8%)	0	0.005^a,b^
Vancomycin /linezolid at bronchoscopy	2 (33.3%)	2 (6.3%)	0	0.073
Detected pathogen				
Bacteria	2 (33.3%)	3 (9.1%)	0	0.262
Atypical	1 (16.7%)	10 (30.3%)	3 (23.1%)	0.737
Virus	2 (33.3%)	11 (33.3%)	4 (30.8%)	0.828
Fungus	0	3 (9.1%)	1 (7.7%)	0.836
Unknown	2	12	7	
Complications & Outcome				
Hypoxemia	6 (100%)	13 (39.4%)	3 (23.1%)	0.005^a,b^
IPPV	3 (50%)	5 (15.2%)	0	0.022^b^
AKI	2 (33.3%)	2 (6.1%)	0	0.07
30-day mortality	1 (16.7%)	5 (15.2%)	0	0.333
LOS (days)	25.0 ± 22.3	13.4 ± 6.5	10.5 ± 3.4	0.092

Data are reported as n (%) for categorical data, mean (±SD) for parametrically distributed data, or median (interquartile range) for nonparametrically distributed data. Statistically significant differences in variables amongst three groups are calculated using one-way ANOVA with post-hoc Turkey HSD test or Kruskal-Wallis H test for continuous data; and Fisher’s exact test with a Bonferroni correction for categorical data. ^a^ statistically significance exists between LClus1 and LClus2; ^b^ statistically significance exists between LClus1 and LClus3; ^c^ statistically significance exists between LClus2 and LClus3; ^d^ Sample size is different than above (namely CAP patients only): LClus1, *n* = 6, LClus2, *n* = 33, LClus3, *n* = 13; ^e^ Recent usage is defined as use of antibiotics or corticosteroids < 2 weeks before admission

Abbreviations: *BUN* blood urea nitrogen, *Cr* creatinine, *ALT* alanine transaminase, *AST* aspartate aminotransferase, *CK* creatine kinase, *ALB* albumin, *TC* total cholesterol, *TG* total triglyceride, *WBC* white blood cell, *NLR* neutrophil to lymphocyte ratio, *BALF* bronchoalveolar lavage, *PMN* polymorphonuclear leukocyte, *PCT* procalcitonin, *CRP* C-reactive protein, *ESR* erythrocyte sedimentation rate, *IPPV* invasive positive pressure ventilation, *AKI* acute kidney injury, *LOS* length of hospital stay

### Distinct lipid profiles of the three LClusters

We originally hypothesized that variation in the lipid compositions of each of the three clusters was related to differences in clinical characteristics among patients. However, the high- (LClus1) and the medium- (LClus2) inflammatory response groups had distinct alterations of BALF lipidomes compared to the low-inflammatory response group (LClus3) (Table 4, Fig. 4b). LClus1 was characterized by the highest mean relative abundance of unsaturated FA (including MUFA and PUFA) and CerG2 classes. In contrast, LClus2 exhibited the highest level of SFA and TG classes, and the lowest level of sphingolipids (SM and GM3 classes) and phospholipids (PC, PE, PG, PI, and PS classes). Subsequently, we identified 41 lipid species (Additional file 2: Table S3) that differed amongst all three clusters (ANOVA with Tukey host HSD test, FDR adjusted *p*-value < 0.05). In accordance with the results of lipid class analysis, we observed significant abundant n-3 PUFA (FA 18:3, α-linolenic acid) in LClus1. Two SFA species (FA 16:0 and FA 18:0, comprising 53.62% of the total lipid signal) and 3 TG species were notably increased, while 7 SM, 12 PC, 8 PE, 1 PG, 5 PI, and 2 PS species were significantly decreased in LClus2. Generally, the differential accumulation of lipid species revealed opposite trends in the changes in BALF lipid composition, compared to the low response group (LClus3). These data suggest that different underlying mechanisms may govern lipid regulation in response to the degree of inflammation during CAP.

**Table 4:** Comparisons of lipid subclasses amongst the three clusters

Lipid class	Lipid Sub-Class	Fold change			Kruskal-Wallis adjusted *p*-value	Tendency
		LClus1/LClus3	LClus2/LClus3	LClus1/LClus2		
Acyl carnitines	AcCa	0.67	0.31^***^	2.18	< 0.0005	
Fatty acids	SFA	0.79	1.08^***^	0.73^***^	< 0.0005	Up in LClus2
	MUFA	3.59^***^	1.03	3.48^**^	< 0.0005	Up in LClus1
	PUFA	3.66^**^	0.84	4.34^***^	< 0.0005	Up in LClus1
Sphingolipids	Cer	0.54	0.81	0.66	0.117	
	CerG1	1.10	0.52^***^	2.11	< 0.0005	
	CerG2	8.95^*^	4.58	1.95^**^	0.009	Up in LClus1
	GM3	1.00	0.23^***^	4.46^***^	< 0.0005	Down in LClus2
	SM	1.37	0.49^***^	2.81^***^	< 0.0005	Down in LClus2
	So	1.37	0.49^*^	2.81	0.013	
Neutral lipids	DG	1.19	22.90	0.05	0.136	
	TG	0.82	1.23^***^	0.66^**^	< 0.0005	Up in LClus2
Phospholipids	PC	1.85	0.40^***^	4.67^***^	< 0.0005	Down in LClus2
	PE	2.88	0.68^***^	4.27^***^	< 0.0005	Down in LClus2
	PG	0.95	0.31^***^	3.09^*^	< 0.0005	Down in LClus2
	PI	1.95	0.39^***^	4.95^***^	< 0.0005	Down in LClus2
	PS	1.84	0.61^***^	3.01^*^	< 0.0005	Down in LClus2
	LPC	0.52	0.43^***^	1.20	< 0.0005	
	LPE	0.45	0.45^***^	0.99	< 0.0005	
	LPG	0.79	0.31^***^	2.53	< 0.0005	

Tendency is indicated only when the cluster is differed from all the other clusters. The * depicts a statistically significant difference. * *p* < 0.05; ** *p* < 0.01; *** *p* < 0.001

### Correlations between lipids and clinical indices

**Fig. 5:**
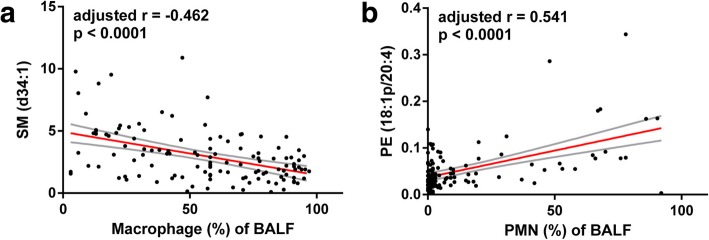
Two lipid species show significantly correlations with BALF cellular components. **a** SM (d34:1) is inversely correlated to macrophage percentages of BALF, adjusted r = − 0.462, *p* < 0.0001. **b** PE (18:1p/20:4) is positively correlated with PMN percentages of BALF, spearman rank r = 0.3639, *p* < 0.0001. Red line, the fitted regression line. Areas within the grey lines, the 95% confidence intervals

Spearman rank correlation analysis was applied to detect whether the differentially accumulating lipids of clusters were correlated with clinical parameters, including percentages of macrophages and neutrophils in BALF, serum ALB, and serum TC levels. When a correlation between lipid levels and clinical parameters was identified, multiple linear regression (MLR) analysis was then conducted using a stepwise method to adjust for the multi-collinearity inherent in lipidomic data (Additional file 2: Table S4, Fig. 5). Finally we identified that SM (d34:1) was negatively related to macrophage percentages in BALF (adjusted r = − 0.462, *p* < 0.0001) and PE (18:1p/20:4) was positively correlated with PMN percentages of BALF (adjusted r = 0.541, *p* < 0.0001). No significant correlations were observed between differential lipid species and ALB or TC, indicating that shifts in the BALF lipidome are related to a local rather than a systemic inflammatory response.

## Discussion

In the current study, we characterized the BALF lipidomes of 120 subjects including 52 CAP patients and 68 controls. We found that in BALF samples from CAP patients, the levels of unsaturated FA, CerG2, and PE classes were increased compared to controls, while the So class was decreased. Using classifications based on clinical-demographic features (regardless of disease severity, age, gender, or pathogen types) with principal component analysis revealed no clear patterns of separation for distinguishing lipid profiles that correlated with any of the clinical features. However, cluster analysis identified three distinct lipid clusters; subjects in different clusters exhibited significant differences in disease severity, the incidence of hypoxemia, the percentages of PMNs and macrophages in BALF, and the serum concentrations of albumin and total cholesterol. We also identified individual lipids at the molecular level (SM d34:1 and PE 18:1p/20:4) that significantly correlated with the phagocytes (PMNs and macrophages) in BALF. Our findings thus suggest that the specific lipid composition in the lower airway may relate to the intensity of the host inflammatory response, rather than clinical and demographic differences, and may contribute to functionally relevant shifts in CAP disease pathogenesis. Based on their appearance in consistent ratios across severely inflamed cases of CAP, SM d34:1 and PE 18:1p/20:4 may serve as reliable diagnostic markers for increased infiltration by macrophage and PMNs, respectively, which accompany local points of tissue inflammation, and thus may serve as potentially useful starting points for development of targeted therapeutics for CAP infection.

Our data showed that patients in different lipid clusters exhibited significantly different degrees of PMNs infiltrations. PMNs and macrophages are major phagocytes that play crucial roles in inflammation, the first line of defense against invaders. Activated tissue macrophages will drive inflammation by recruiting neutrophils and other leukocytes [23]. However, excessive infiltration by PMNs can also result in tissue damage. Furthermore, increases in tissue PMNs can be used as indicators of the degree of local inflammation. The high- (LClus1) and the medium- (LClus2) inflammatory response groups had inversely correlated lipid profiles in comparison to the low response group (LClus3). LClus1, high in PMNs, was characterized by abundant phospholipids (mostly PC and PE species), likely indicating damaged tissue from excessive and destructive lung inflammation, given that phospholipids are major components of all cell membranes.

However, it remains unclear if the prevalent lipids in LCLus1 and LCLus2 are responsible for the elevated inflammation, or if; conversely, the PC and PE phospholipids are produced by inflammation-induced tissue damage. Tisoncik et al. [24] reported significant abundant phospholipids (PC and PE species) in bronchial epithelial cells of ferrets in response to influenza virus infection. They speculate that the lipid mediators derived from phospholipid arachidonic acid (FA 20:4) reservoirs may contribute to tissue damage during pandemic H1N1 influenza virus infection. Our finding that PE 18:1p/20:4 is positively correlated with PMN percentages in BALF may support their hypothesis. The 20:4 side-chain in PE 18:1p/20:4 can be cleaved to form arachidonic acid, the precursor to eicosanoids converted by the cyclooxygenase-2 (COX-2) enzyme. Increased production of arachidonic acid-derived pro-inflammatory prostaglandin and leukotriene levels [25] can induce PMN infiltration [26, 27], enhance inflammatory responses, and finally lead to tissue damage. In addition, although sphingolipid signal transduction pathways have been known to play an important role in the regulation of growth and survival pathways in macrophages [28], the exact role of SM (d34:1) still requires future research.

Our data shows that fatty acids are notably involved in CAP. First, they are the most abundant lipids detected in the BALF lipidome in our study. Furthermore, BALF levels of unsaturated FA (including MUFA and PUFA) are significantly elevated in CAP and the high-inflammatory response group (LClus1); while levels of SFA are increased in the medium-inflammatory response group (LClus2). Third, several MUFAs (FA 16:1, FA 18:1, and FA 20:1) could be effective markers for SCAP. Moreover, FA 18:3, an n-3 PUFA, is notably elevated in the high-inflammatory response group (LClus1).

Previous studies have revealed that both levels and compositions of FAs are associated with inflammation [29]. Generally, SFAs are considered as pro-inflammatory lipid mediators as they induce the inflammation by mimicking the actions of lipopolysaccharide (LPS) [30], activating the NLRP3 inflammasome [31], and inducing NFΚB signaling [32], etc. In contrast, PUFAs are commonly considered as anti-inflammatory lipid mediators as they can inhibit inflammasome activation [33] and can inhibit endothelial cell activation following exposure to LPS [34]. In addition, specialized pro-resolving lipid (SPM) mediators (the resolvins and the protectins/neuroprotectins) derived from n-3 PUFA [35] reveal their role as anti- and pro-resolving inflammation as well as enhancing microbial clearance [8]. Unexpectedly, our data suggested the opposite tendencies of pro- and anti-inflammatory lipid alternations in either CAP patients or patients in LClus1. Recently Sun and colleagues report that same pattern of accumulation of serum palmitic acid (FA 16:0) during H7N9 pneumonia. The levels of palmitic acid decreased while the clinical conditions became more severe, and increased as the disease was ameliorated [36]. Schmidt et al. also found that PUFA increased approximately 3-fold in BALF of adult respiratory disease syndrome (ARDS) patients [37]. In light of our own data, which agrees with the findings of Schmidt and Sun, we are compelled to reconsider the pro- and anti-inflammatory designations assigned to bioactive lipids, since their proposed activity is not supported by our HPLC-MS data in CAP patients. We speculate that the role of bioactive lipids, such as PUFAs in mitigating inflammation, is context dependent and may be influenced by other factors that fall outside the scope of this study.

TG and SM classes, the top 2 and the top 3 lipid sub-classes of the total BALF lipidome, respectively, show no difference between CAP and controls, and no difference between the LClus1 high-inflammatory response group and the LClus3 low-inflammatory response group. Previous studies have reported hypertriglyceridemia during infection, and different types of infection (bacterial, viral, or fungal) result in similar effects on triglyceride metabolism [38–41]. Infection-associated hypertriglyceridemia is induced via increased hepatic lipoprotein production and/or decreased lipoprotein catabolism [42]. However, some clinical studies came to the opposite conclusion, partially supporting our observations, in that lower or no significantly changes in triglyceride levels were observed in critically ill and severely infected patients [43, 44]. Moreover, Kaysen and colleagues reported an inverse association between serum triglyceride levels and all-cause mortality (HR: 0.93, 95% CI: 0.90–0.96) [45].

Likewise, inflammation induces SM hydrolysis, tending to decrease the SM mass and lead to ceramide accumulation [46]. Nonetheless, the enhanced de novo sphingolipid biosynthesis during inflammation can lead to the formation of ceramide and subsequent the formation of sphingomyelin [47]. Which might be the most plausible explanation for the sustained SM levels in LClus1.

Together with previous studies, our work suggests that distinct lipid profiles repeat over numbers of patients, accompanied by different airway inflammatory status and disease severity, although host-lipidome is considered uniformly detrimental to patients with the same disease. Whether the observed lipidome changes are a cause or a consequence of the development of pneumonia or merely coincide with disease status remains a question. Larger cohorts of patients are necessary to sufficiently power studies examining lipid compositions and quantifications, their inflammatory status, and clinical implications. Further longitudinal studies would advance understanding of temporal changes in lipids prior to disease onset.

## Conclusion

The global profiling analysis of CAP uncovered complex lipidomic responses to infection, with the levels of mediators involved in both pro- and anti-inflammatory processes. Thus arguing for the potential need to tailor therapy on the specific disturbed lipid profiles and related inflammatory status exhibited by the subjects.

## Additional files

Additional file 1: Supplementary Methods. (DOCX 13 kb)

Additional file 2: **Tables S1.** Summary of unique lipid species, by class, identified using LC-MS. **Table S2.** Thirty-three lipid species differentiated SCAP from controls. **Table S3.** Forty-one lipid species differed amongst three lipid clusters (LClus). **Table S4.** Correlation matrix of differential lipids of clusters and phagocyte percentages of BALF. (ZIP 123 kb)

Additional file 3: **Figure S1.** Receiver operating characteristics (ROC) curves for FA (16:1), FA (18:1), FA (20:1), and FA (24:0) show the abilities to discriminate SCAP from NSCAP. **Figure S2.** Result of the elbow method to determine optimum number of clusters (*k* = 3). (ZIP 287 kb)

## Abbreviations

AcCa: Acylcarnitine
Cer: Ceramide
CerG1: Glucosylceramide
ChE: Cholesterol ester
DG: Diglyceride
FA: Fatty acid
GM3: Monosialotetrahexosylganglioside-3
LacCer: Lactosylceramide
LPC: Lysophosphatidylcholine
LPE: Lysophosphatidylethanolamine
LPG: Lysophosphatidylglycerol
PC: Phosphatidylcholine
PE: Phosphatidylethanolamine
PG: Phosphatidylglycerol
PI: Phosphatidylinositol
PS: Phosphatidylserine
SM: Sphingomyelin
So: Sphingosine
TG: Triglyceride
