# Supplementary material for: Inflammatory responses relate to distinct bronchoalveolar lavage lipidome in community-acquired pneumonia patients: a pilot study
Source: Respir Res. 2019 May 2;20:82. doi: 10.1186/s12931-019-1028-8 (PMC6498485; doi:10.1186/s12931-019-1028-8)
Supplement: Supplementary file 1 — Supplementary Methods. (DOCX 13 kb) [file 12931_2019_1028_MOESM1_ESM.docx]

Supplementary Method

HPLC methods for lipids were as follows: Mobile phase A was prepared by dissolving 0.77 g of ammonium acetate to 400 mL of HPLC-grade water, followed by adding 600 mL of HPLC-grade acetonitrile. Mobile phase B was prepared by mixing 100 mL of acetonitrile with 900 mL isopropanol. The gradient was as below: 0 min, 37% B; 1.5 min, 37% B; 4 min, 45% B; 5 min, 52% B; 8 min, 58% B; 11 min, 66% B; 14 min, 70% B; 18 min, 75% B; 20 min, 98% B; 22 min, 98% B; 22.1 min 37% B; 25 min, 37% B. The detailed MS parameters are as follows: spray voltage, 3.2 kV for positive and 2.8 kV for negative; capillary temperature, 320 °C; aux gas flow rate (arb), 10; Mass range (m/z), 240–2000 for positive and 200–2000 for negative.
