# Supplementary material for: Inflammatory responses relate to distinct bronchoalveolar lavage lipidome in community-acquired pneumonia patients: a pilot study
Source: Respir Res. 2019 May 2;20:82. doi: 10.1186/s12931-019-1028-8 (PMC6498485; doi:10.1186/s12931-019-1028-8)
Supplement: Supplementary file 2 — Tables S1. Summary of unique lipid species, by class, identified using LC-MS. Table S2. Thirty-three lipid species differentiated SCAP from controls. Table S3. Forty-one lipid species differed amongst three lipid clusters (LClus). Table S4. Correlation matrix of differential lipids of clusters and phagocyte percentages of BALF. (ZIP 123 kb) [file 12931_2019_1028_MOESM2_ESM.zip › Additional file 2. Table S1. Summary of unique lipid species.pdf]

Table S1. Summary of unique lipid species, by class, identified using LC-MS

Lipid Class	Lipid Sub-Classes	Lipid Search	Count of Unique Species	Lipid species	% of Total Lipid Singal	Ion Polarity	Main Ion	ESI+ mode		ESI- mode	
								RT	m/z	RT	m/z
Acylcarnitines		AcCa	2	AcCa (16:0)	0.013%	pos	+H	2.56	400.34		
				AcCa (18:1)	0.013%	pos	+H	2.74	426.36		
Fatty acids		FA	16	FA (14:0)	0.296%	neg	-H			2.51	227.20
				FA (15:0)	0.202%	neg	-H			3.21	241.22
				FA (16:0)	25.960%	neg	-H			4.1	255.23
				FA (16:1)	0.191%	neg	-H			2.76	253.22
				FA (17:0)	0.275%	neg	-H			5.29	269.25
				FA (18:0)	27.662%	neg	-H			6.59	283.26
				FA (18:1)	1.127%	neg	-H			4.55	281.25
				FA (18:2)	0.565%	neg	-H			3.06	279.23
				FA (18:3)	0.039%	neg	-H			2.28	277.22
				FA (20:0)	0.463%	neg	-H			8.78	311.30
				FA (20:1)	0.031%	neg	-H			6.91	309.28
				FA (20:4)	0.172%	neg	-H			2.79	303.23
				FA (22:4)	0.023%	neg	-H			4.13	331.26
				FA (22:5)	0.016%	neg	-H			3.06	329.25
				FA (22:6)	0.030%	neg	-H			2.39	327.23
				FA (24:0)	0.100%	neg	-H			12.89	367.36
Sphingolipids	Ceramide	Cer	7	Cer (d18:1/16:0)	0.358%	pos, neg	+H, +CH3COO	14.78	538.5194	14.78	596.526
				Cer (d18:1/18:0)	0.168%	pos, neg	+H, +CH3COO	16.39	566.5507	16.4	624.5573
				Cer (d18:1/20:0)	0.052%	pos, neg	+H, +CH3COO	18.06	594.582	18.07	652.5886
				Cer (d18:1/22:0)	0.056%	pos, neg	+H, +CH3COO	19.76	622.6133	19.77	680.6199
				Cer (d18:1/24:0)	0.159%	pos, neg	+H, +CH3COO	21.36	650.6446	21.35	708.6512
				Cer (d18:1/24:1)	0.098%	pos, neg	+H, +CH3COO	19.59	648.6289	19.59	706.6355
				Cer (d18:1/24:2)	0.036%	pos	+H	18.07	646.6133		
	Glucosylcera mide	CerG1	5	CerG1 (d18:1/16:0)	0.075%	pos	+H	13.46	700.5722		
				CerG1 (d18:1/22:0)	0.059%	pos	+H	18.11	784.6661		
				CerG1 (d18:1/23:0)	0.018%	pos	+H	18.93	798.6818		
				CerG1 (d18:1/24:0)	0.090%	pos	+H	19.73	812.6974		
				CerG1 (d18:1/24:1)	0.076%	pos	+H	17.95	810.6818		
	Lactosylcera mide	CerG2	3	CerG2 (d18:1/16:0)	0.101%	pos	+H	12.82	862.625		
				CerG2 (d18:1/22:0)	0.026%	pos	+H	17.34	946.7189		
				CerG2 (d18:1/24:1)	0.126%	pos, neg	+H, +CH3COO	17.19	972.7346	17.19	970.72
	Monosialotetr ahexosylgangl	GM3	1								
				GM3 (d18:1/24:1)	0.013%	pos	+H	13.8	1263.83		
	Sphingomyeli n	SM	15	SM (d15:0/18:1)	0.035%	pos	+H	12.06	689.5592		
				SM (d16:0/18:1)	1.782%	pos	+H	12.91	703.5749		
				SM (d16:0/24:1)	0.835%	pos	+H	17.75	787.6688		
				SM (d16:0/24:2)	0.190%	pos	+H	16.22	785.6531		
				SM (d16:0/25:1)	0.260%	pos	+H	18.58	801.6844		
				SM (d16:0/25:2)	0.040%	pos	+H	16.73	799.6688		
				SM (d16:0/26:1)	1.160%	pos, neg	+H, +CH3COO	19.43	815.7001	19.56	873.7066
				SM (d16:0/26:2)	1.888%	pos, neg	+H, +CH3COO	17.5	813.6844	17.55	871.691
				SM (d16:0/26:3)	0.593%	pos, neg	+H, +CH3COO	16.09	811.6688	16.1	869.6753
				SM (d16:1/16:0)	0.048%	pos, neg	+H, +CH3COO	11.16	675.5436	11.16	733.5501
				SM (d18:1/18:0)	0.348%	pos, neg	+H, +CH3COO	14.49	731.6062	14.45	789.6127
				SM (d18:1/18:1)	0.040%	pos	+H	13.09	729.5905		
				SM (d18:1/26:4)	0.551%	pos	+H	17.54	835.6688		
				SM (d20:1/14:0)	0.129%	neg	+CH3COO			12.9	761.5814
				SM (d22:2/19:1)	0.012%	neg	+CH3COO			16.08	855.6597
	Sphingosine	So	4	So (d16:1)	0.051%	pos	+H	3.28	272.2584		
				So (d18:0)	0.685%	pos	+H	2.99	302.3054		
				So (d18:1)	0.029%	pos	+H	2.99	300.2897		
				So (d20:1)	0.048%	pos	+H	7.85	328.321		
Neutral lipids	Diglyceride	DG	3	DG (18:0/16:0)	0.939%	pos	+NH4	19.06	614.5718		
				DG (18:0/18:0)	0.853%	pos	+NH4	20.76	642.6031		
				DG (18:1/18:2)	0.087%	pos	+NH4	16.19	636.5562		
	Triglyceride	TG	18	TG (14:0/18:2/18:2)	0.310%	pos	+NH4	22.65	844.7389		
				TG (15:0/16:0/18:1)	0.725%	pos	+NH4	23.24	836.7702		
				TG (16:0/14:0/16:0)	0.900%	pos	+NH4	23.14	796.7389		
				TG (16:0/14:0/16:1)	1.479%	pos	+NH4	22.89	794.7232		
				TG (16:0/16:0/16:0)	1.026%	pos	+NH4	23.35	824.7702		
				TG (16:0/16:0/16:1)	1.811%	pos	+NH4	23.13	822.7545		
				TG (16:0/16:0/18:1)	1.447%	pos	+NH4	23.34	850.7858		
				TG (16:0/16:0/18:2)	2.762%	pos	+NH4	23.13	848.7702		
				TG (16:0/17:1/18:1)	0.477%	pos	+NH4	23.23	862.7858		
				TG (16:0/18:1/18:1)	2.368%	pos	+NH4	23.32	876.8015		
				TG (16:0/18:1/18:2)	3.070%	pos	+NH4	23.13	874.7858		
				TG (16:0/18:2/18:2)	1.662%	pos	+NH4	22.91	872.7702		
				TG (16:1/16:1/18:1)	1.213%	pos	+NH4	22.89	846.7545		
				TG (16:1/18:2/18:2)	0.479%	pos	+NH4	22.7	870.7545		
				TG (18:0/18:1/18:1)	0.545%	pos	+NH4	23.51	904.8328		
				TG (18:1/18:1/18:1)	0.918%	pos	+NH4	23.32	902.8171		
				TG (18:1/18:1/18:2)	1.332%	pos	+NH4	23.13	900.8015		
				TG (18:1/18:2/18:2)	1.245%	pos	+NH4	22.91	898.7858		
Phospholipids	Phosphatidylc holine	PC	24								
				PC (14:0/18:2)	0.018%	neg	+CH3COO			11.7	788.5447
				PC (15:0/16:0)	0.038%	neg	+CH3COO			13.64	778.5604
				PC (16:0/14:0)	0.177%	neg	+CH3COO			12.85	764.5447
				PC (16:0/16:0)	1.972%	neg	+CH3COO			14.36	792.576
				PC (16:0/16:1)	0.301%	neg	+CH3COO			13	790.5604
				PC (16:0/17:0)	0.022%	neg	+CH3COO			14.84	806.5917
				PC (16:0/18:1)	0.533%	neg	+CH3COO			14.46	818.5917
				PC (16:0/18:2)	0.468%	neg	+CH3COO			13.28	816.576
				PC (16:0/18:3)	0.014%	neg	+CH3COO			12.51	814.5604

			PC (16:0/20:4)	0.088%	neg	+CH3COO			12.99	840.576
			PC (16:0/22:6)	0.019%	neg	+CH3COO			12.56	864.576
			PC (16:0e/16:0)	0.033%	neg	+CH3COO			15.41	778.5968
			PC (16:0p/16:0)	0.016%	neg	+CH3COO			15.14	776.5811
			PC (16:1/18:2)	0.014%	neg	+CH3COO			11.85	814.5604
			PC (17:0/18:1)	0.006%	neg	+CH3COO			15.22	832.6073
			PC (18:0/16:0)	0.142%	neg	+CH3COO			15.91	820.6073
			PC (18:0/18:1)	0.053%	neg	+CH3COO			16.06	846.623
			PC (18:0/18:2)	0.229%	neg	+CH3COO			14.79	844.6073
			PC (18:0/20:3)	0.011%	neg	+CH3COO			15.06	870.623
			PC (18:0/20:4)	0.047%	neg	+CH3COO			14.47	868.6073
			PC (18:0/22:6)	0.010%	neg	+CH3COO			14.03	892.6073
			PC (18:0e/20:4)	0.007%	neg	+CH3COO			15.44	854.6281
			PC (18:0p/16:0)	0.028%	neg	+CH3COO			15.44	804.6124
			PC (18:0p/20:4)	0.018%	neg	+CH3COO			13.98	852.6124
Phosphatidylethanolamine	PE	18								
			PE (16:0/16:0)	0.003%	neg	-H			14.72	690.5079
			PE (16:0/18:1)	0.041%	neg	-H			14.79	716.5236
			PE (16:0/18:2)	0.025%	neg	-H			13.63	714.5079
			PE (16:0/20:4)	0.009%	neg	-H			13.39	738.5079
			PE (16:0p/18:1)	0.054%	neg	-H			15.59	700.5287
			PE (16:0p/18:2)	0.012%	neg	-H			14.37	698.513
			PE (16:0p/20:4)	0.076%	neg	-H			14.05	722.513
			PE (16:0p/22:4)	0.021%	neg	-H			15.08	750.5443
			PE (18:0/18:1)	0.057%	neg	-H			16.3	744.5549
			PE (18:0/18:2)	0.078%	neg	-H			15.06	742.5392
			PE (18:0/20:4)	0.031%	neg	-H			14.8	766.5392
			PE (18:0p/18:1)	0.031%	neg	-H			17.15	728.56
			PE (18:0p/18:2)	0.017%	neg	-H			15.9	726.5443
			PE (18:0p/20:4)	0.078%	neg	-H			15.53	750.5443
			PE (18:0p/22:6)	0.023%	neg	-H			15.04	774.5443
			PE (18:1/18:1)	0.080%	neg	-H			14.85	742.5392
			PE (18:1/18:2)	0.048%	neg	-H			13.72	740.5236
			PE (18:1p/20:4)	0.039%	neg	-H			14.11	748.5287
Phosphatidylglycerol	PG	12								
			PG (16:0/16:0)	0.056%	neg	-H			12.01	721.5025
			PG (16:0/16:1)	0.016%	neg	-H			10.77	719.4869
			PG (16:0/18:1)	0.213%	neg	-H			12.1	747.5182
			PG (16:0/18:2)	0.081%	neg	-H			11.05	745.5025
			PG (16:0/20:4)	0.015%	neg	-H			10.81	769.5025
			PG (16:0/22:6)	0.006%	neg	-H			10.44	793.5025
			PG (18:0/16:0)	0.019%	neg	-H			13.27	749.5338
			PG (18:0/18:1)	0.118%	neg	-H			13.35	775.5495
			PG (18:0/18:2)	0.174%	neg	-H			12.19	773.5338
			PG (18:1/18:1)	0.031%	neg	-H			11.74	773.5338
			PG (18:1/18:2)	0.026%	neg	-H			10.6	771.5182
			PG (18:2/18:2)	0.012%	neg	-H			10.15	769.5025
Phosphatidylinositol	PI	10								
			PI (16:0/16:0)	0.007%	neg	-H			11.67	809.5186
			PI (16:0/18:1)	0.076%	neg	-H			11.8	835.5342
			PI (16:0/22:6)	0.009%	neg	-H			10.13	881.5186
			PI (18:0/16:0)	0.006%	neg	-H			12.98	837.5499
			PI (18:0/18:1)	0.113%	neg	-H			13.08	863.5655
			PI (18:0/20:3)	0.008%	neg	-H			12.41	887.5655
			PI (18:0/20:4)	0.051%	neg	-H			11.88	885.5499
			PI (18:0/22:6)	0.006%	neg	-H			11.5	909.5499
			PI (18:1/18:1)	0.161%	neg	-H			11.9	861.5499
			PI (18:1/20:4)	0.016%	neg	-H			10.57	883.5342
phatidylserine	PS	3								
			PS (18:0/18:1)	0.060%	neg	-H			13.35	788.5447
			PS (18:0/18:2)	0.023%	neg	-H			12.32	786.5291
			PS (18:0/20:4)	0.009%	neg	-H			12.09	810.5291
Lysophosphatidylcholine	LPC	3	LPC (16:0)	0.383%	pos, neg	+H, +CH3COO	2.73	496.3398	2.75	554.3464
			LPC (18:0)	0.077%	pos, neg	+H, +CH3COO	4.44	524.3711	4.49	582.3777
			LPC (18:1)	0.095%	pos, neg	+H, +CH3COO	2.92	522.3554	2.95	580.362
Lysophosphatidylethanolamine	LPE	3								
			LPE (16:0)	0.013%	pos, neg	+H, -H	2.89	454.2928	2.9	452.2783
			LPE (18:0)	0.023%	pos, neg	+H, -H	4.73	482.3241	4.76	480.3096
			LPE (18:1)	0.046%	pos, neg	+H, -H	3.09	480.3085	3.11	478.2939
Lysophosphatidylglycerol	LPG	3								
			LPG (16:0)	0.002%	neg	-H			2.07	483.2729
			LPG (18:0)	0.001%	neg	-H			3.16	511.3042
			LPG (18:1)	0.005%	neg	-H			2.19	509.2885
