# Supplementary material for: Inflammatory responses relate to distinct bronchoalveolar lavage lipidome in community-acquired pneumonia patients: a pilot study
Source: Respir Res. 2019 May 2;20:82. doi: 10.1186/s12931-019-1028-8 (PMC6498485; doi:10.1186/s12931-019-1028-8)
Supplement: Supplementary file 2 — Tables S1. Summary of unique lipid species, by class, identified using LC-MS. Table S2. Thirty-three lipid species differentiated SCAP from controls. Table S3. Forty-one lipid species differed amongst three lipid clusters (LClus). Table S4. Correlation matrix of differential lipids of clusters and phagocyte percentages of BALF. (ZIP 123 kb) [file 12931_2019_1028_MOESM2_ESM.zip › Additional file 2. Table S2. Lipids discriminating CAP from controls.docx]

Table S2. Thirty-three lipid species differentiated SCAP from Controls.

| **Regulation trend** | **Confirmed lipid species** | **Adduct** | **m/z** | **RT** | **RSD-QC** | **% of total lipids** | **Fold change** | | | **FDR adjusted p-value** |
| --- | --- | --- | --- | --- | --- | --- | --- | --- | --- | --- |
|  |  |  |  |  |  |  | **SCAP/Control** | **NSCAP/Control** | **SCAP/NSCAP** |  |
| SCAP < NSCAP < Control | FA (16:0) | -H | 255.2324 | 4.1 | 2.8% | 25.960% | 0.90 | 0.97 | 0.93 | 3.50E-04 |
| SCAP > NSCAP > Control | FA (18:1) | -H | 281.2481 | 4.55 | 8.6% | 1.127% | 2.19 | 1.11 | 1.97 | 3.77E-05 |
|  | FA (18:2) | -H | 279.2324 | 3.06 | 6.2% | 0.565% | 2.30 | 1.14 | 2.01 | 3.07E-04 |
|  | PC (16:0/18:1) | +CH3COO | 818.5917 | 14.46 | 3.3% | 0.533% | 1.94 | 1.12 | 1.74 | 1.23E-03 |
|  | PC (16:0/18:2) | +CH3COO | 816.576 | 13.28 | 3.2% | 0.468% | 1.73 | 1.06 | 1.64 | 2.30E-02 |
|  | PC (18:0/18:2) | +CH3COO | 844.6073 | 14.79 | 5.5% | 0.229% | 2.21 | 1.19 | 1.86 | 3.78E-04 |
|  | FA (16:1) | -H | 253.2168 | 2.76 | 19.4% | 0.191% | 1.73 | 1.21 | 1.44 | 5.78E-04 |
|  | SM (d34:1) | +CH3COO | 761.5814 | 12.9 | 2.4% | 0.129% | 4.18 | 2.45 | 1.71^a^ | 4.65E-05 |
|  | FA (24:0) | -H | 367.3576 | 12.89 | 11.1% | 0.100% | 1.35 | 1.11 | 1.21 | 6.41E-04 |
|  | PC (16:0/20:4) | +CH3COO | 840.576 | 12.99 | 3.4% | 0.088% | 2.59 | 1.30 | 2.00 | 1.79E-04 |
|  | PE (18:1/18:1) | -H | 742.5392 | 14.85 | 5.5% | 0.080% | 1.55 | 1.02 | 1.52 | 2.12E-02 |
|  | PE (18:0/18:2) | -H | 742.5392 | 15.06 | 5.5% | 0.078% | 1.58 | 1.03 | 1.53 | 1.82E-02 |
|  | PE (16:0p/20:4) | -H | 722.513 | 14.05 | 4.5% | 0.076% | 2.76 | 1.66 | 1.67^a^ | 2.83E-05 |
|  | PC (18:0/18:1) | +CH3COO | 846.623 | 16.06 | 8.6% | 0.053% | 3.64 | 1.30 | 2.79 | 8.73E-04 |
|  | PI (18:0/20:4) | -H | 885.5499 | 11.88 | 1.2% | 0.051% | 3.30 | 1.53 | 2.17 | 5.41E-05 |
|  | PC (18:0/20:4) | +CH3COO | 868.6073 | 14.47 | 4.4% | 0.047% | 2.99 | 1.48 | 2.01 | 4.26E-05 |
|  | PE (16:0/18:1) | -H | 716.5236 | 14.79 | 5.4% | 0.041% | 1.72 | 1.09 | 1.58 | 4.78E-03 |
|  | SM (d36:2) | +H | 729.5905 | 13.09 | 3.5% | 0.040% | 3.06 | 1.46 | 2.10 | 1.08E-03 |
|  | PE (18:1p/20:4) | -H | 748.5287 | 14.11 | 4.7% | 0.039% | 2.63 | 1.54 | 1.70 | 3.97E-05 |
|  | PE (18:0/20:4) | -H | 766.5392 | 14.8 | 5.1% | 0.031% | 2.45 | 1.45 | 1.70 | 4.65E-05 |
|  | FA (20:1) | -H | 309.2794 | 6.91 | 6.5% | 0.031% | 2.62 | 1.34 | 1.95 | 1.86E-05 |
|  | PC (18:0p/16:0) | +CH3COO | 804.6124 | 15.44 | 5.3% | 0.028% | 4.58 | 2.40 | 1.91 | 5.17E-05 |
|  | PE (16:0p/22:4) | -H | 750.5443 | 15.08 | 3.9% | 0.021% | 3.28 | 1.89 | 1.74 | 6.19E-05 |
|  | PC (16:0/22:6) | +CH3COO | 864.576 | 12.56 | 3.4% | 0.019% | 4.56 | 1.87 | 2.44 | 6.19E-05 |
|  | SM (d41:3) | +CH3COO | 855.6597 | 16.08 | 7.1% | 0.012% | 4.87 | 2.21 | 2.20 | 2.62E-05 |
|  | PC (18:0/20:3) | +CH3COO | 870.623 | 15.06 | 4.3% | 0.011% | 3.44 | 2.04 | 1.69^a^ | 6.19E-05 |
|  | PC (18:0/22:6) | +CH3COO | 892.6073 | 14.03 | 4.5% | 0.010% | 4.97 | 2.29 | 2.17 | 6.19E-05 |
|  | PI (18:0/20:3) | -H | 887.5655 | 12.41 | 6.4% | 0.008% | 2.79 | 1.71 | 1.63 | 1.61E-04 |
|  | PI (18:0/22:6) | -H | 909.5499 | 11.5 | 2.2% | 0.006% | 2.11 | 1.19 | 1.78 | 3.40E-03 |
|  | PE (16:0/16:0) | -H | 690.5079 | 14.72 | 5.0% | 0.003% | 2.04 | 1.24 | 1.64 | 6.39E-03 |
| SCAP > Control > NSCAP | PI (18:1/20:4) | -H | 883.5342 | 10.57 | 2.6% | 0.016% | 1.62 | 0.91 | 1.78 | 4.10E-02 |
|  | PE (16:0/20:4) | -H | 738.5079 | 13.39 | 4.4% | 0.009% | 2.38 | 0.99 | 2.41 | 2.95E-03 |
|  | PI (16:0/16:0) | -H | 809.5186 | 11.67 | 2.6% | 0.007% | 1.52 | 0.98 | 1.55 | 1.09E-02 |

Abbreviations: RSD, relative standard deviation; QC, quality control; FDR, false discovery rate. Statistically significant differences in variables amongst three groups were calculated using one-way ANOVA with post-hoc Turkey HSD test. Significantly altered lipid species were ranked by their proportions. ^a^ depicts a statistically significant difference between SCAP and NSCAP.
