# Supplementary material for: Inflammatory responses relate to distinct bronchoalveolar lavage lipidome in community-acquired pneumonia patients: a pilot study
Source: Respir Res. 2019 May 2;20:82. doi: 10.1186/s12931-019-1028-8 (PMC6498485; doi:10.1186/s12931-019-1028-8)
Supplement: Supplementary file 2 — Tables S1. Summary of unique lipid species, by class, identified using LC-MS. Table S2. Thirty-three lipid species differentiated SCAP from controls. Table S3. Forty-one lipid species differed amongst three lipid clusters (LClus). Table S4. Correlation matrix of differential lipids of clusters and phagocyte percentages of BALF. (ZIP 123 kb) [file 12931_2019_1028_MOESM2_ESM.zip › Additional file 2. Table S3. Lipid sepcies differed amongst three LClusters.docx]

| **Tendency** | **Identified lipid species** | **Adduct** | **RT** | **m/z** | **RSD-QC** | **% of total lipids** | **Fold change** | | | **FDR adjusted p-value** |
| --- | --- | --- | --- | --- | --- | --- | --- | --- | --- | --- |
|  |  |  |  |  |  |  | **LClus1/LClus2** | **LClus1/LClus3** | **LClus2/LClus3** |  |
| LClus1<LClus3<LClus2 | TG (16:0/14:0/16:0) | +NH4 | 23.14 | 796.7389 | 13.23% | 0.90% | 0.28 | 0.37 | 1.3 | 4.29E-10 |
|  | TG (16:0/16:0/16:1) | +NH4 | 23.13 | 822.7545 | 8.95% | 1.81% | 0.33 | 0.39 | 1.19 | 2.03E-05 |
|  | TG (16:0/16:0/18:2) | +NH4 | 23.13 | 848.7702 | 3.61% | 2.76% | 0.50 | 0.62 | 1.23 | 3.65E-05 |
|  | FA (16:0) | -H | 4.1 | 255.2324 | 2.81% | 25.96% | 0.73 | 0.77 | 1.05 | 2.99E-15 |
|  | FA (18:0) | -H | 6.59 | 283.2637 | 5.04% | 27.66% | 0.73 | 0.81 | 1.11 | 2.54E-21 |
| LClus1>LClus3>LClus2 | FA (18:3) | -H | 2.28 | 277.2168 | 7.76% | 0.04% | 4. 3 | 3.02 | 0.70 | 4.45E-14 |
|  | SM (d34:1) | +H | 12.91 | 703.5749 | 2.70% | 1.78% | 2.94 | 1.92 | 0.65 | 2.55E-08 |
|  | SM (d40:2) | +H | 16.22 | 785.6531 | 2.33% | 0.19% | 5.8 | 3.09 | 0.53 | 1.06E-15 |
|  | SM (d41:2) | +H | 16.73 | 799.6688 | 3.58% | 0.04% | 4.68 | 2.32 | 0.5 | 5.8E-09 |
|  | SM (d42:2) | +H | 17.5 | 813.6844 | 2.91% | 1.89% | 2.97 | 1.38 | 0.46 | 4.74E-20 |
|  | SM (d42:3) | +H | 16.09 | 811.6688 | 2.53% | 0.59% | 3.93 | 2.24 | 0.57 | 1.16E-16 |
|  | SM (d36:1) | +H | 14.49 | 731.6062 | 3.34% | 0.35% | 2.52 | 1.4 | 0.55 | 4.9E-14 |
|  | SM (d44:5) | +H | 17.54 | 835.6688 | 4.45% | 0.55% | 2.92 | 1.35 | 0.46 | 1.91E-19 |
|  | PC (14:0/18:2) | +CH3COO | 11.7 | 788.5447 | 4.53% | 0.02% | 7.49 | 2.8 | 0.37 | 3.09E-13 |
|  | PC (16:0/14:0) | +CH3COO | 12.85 | 764.5447 | 2.93% | 0.18% | 4.03 | 1.48 | 0.37 | 3.01E-13 |
|  | PC (16:0/16:1) | +CH3COO | 13 | 790.5604 | 3.34% | 0.30% | 4.66 | 1.67 | 0.38 | 4.38E-21 |
|  | PC (16:0/18:1) | +CH3COO | 14.46 | 818.5917 | 3.31% | 0.53% | 6.09 | 2.66 | 0.44 | 6.33E-35 |
|  | PC (16:0/18:2) | +CH3COO | 13.28 | 816.576 | 3.18% | 0.47% | 6.40 | 2.61 | 0.41 | 3.85E-28 |
|  | PC (16:0/18:3) | +CH3COO | 12.51 | 814.5604 | 2.78% | 0.01% | 5.41 | 2.54 | 0.47 | 7.24E-10 |
|  | PC (16:0/20:4) | +CH3COO | 12.99 | 840.576 | 3.44% | 0.09% | 6.87 | 3.79 | 0.55 | 7.3E-23 |
|  | PC (16:0p/16:0) | +CH3COO | 15.14 | 776.5811 | 13.02% | 0.02% | 5.77 | 2.24 | 0.39 | 5.57E-17 |
|  | PC (16:1/18:2) | +CH3COO | 11.85 | 814.5604 | 5.33% | 0.01% | 7.08 | 2.64 | 0.37 | 5.02E-17 |
|  | PC (17:0/18:1) | +CH3COO | 15.22 | 832.6073 | 4.23% | 0.01% | 6.64 | 2.60 | 0.39 | 9.76E-24 |
|  | PC (18:0/18:1) | +CH3COO | 16.06 | 846.623 | 8.56% | 0.05% | 18.32 | 5.19 | 0.28 | 2.25E-27 |
|  | PC (18:0/18:2) | +CH3COO | 14.79 | 844.6073 | 5.50% | 0.23% | 6.49 | 3.47 | 0.53 | 6.35E-32 |
|  | PE (16:0/16:0) | -H | 14.72 | 690.5079 | 5.00% | 0.00% | 6.42 | 2.45 | 0.38 | 8.09E-20 |
|  | PE (16:0/18:1) | -H | 14.79 | 716.5236 | 5.36% | 0.04% | 4.14 | 2.11 | 0.51 | 3.79E-18 |
|  | PE (16:0/18:2) | -H | 13.63 | 714.5079 | 4.14% | 0.02% | 4.37 | 1.82 | 0.42 | 7.09E-22 |
|  | PE (16:0/20:4) | -H | 13.39 | 738.5079 | 4.38% | 0.01% | 14.08 | 2.91 | 0.21 | 9.09E-31 |
|  | PE (18:0/18:2) | -H | 15.06 | 742.5392 | 5.53% | 0.08% | 3.80 | 2.1 | 0.55 | 8.18E-16 |
|  | PE (18:0/20:4) | -H | 14.8 | 766.5392 | 5.06% | 0.03% | 4.99 | 3.31 | 0.66 | 3.5E-18 |
|  | PE (18:1/18:1) | -H | 14.85 | 742.5392 | 5.47% | 0.08% | 3.72 | 2.09 | 0.56 | 1.46E-15 |
|  | PE (18:1p/20:4) | -H | 14.11 | 748.5287 | 4.65% | 0.04% | 5.21 | 3.66 | 0.70 | 1.9E-18 |
|  | PG (18:1/18:2) | -H | 10.6 | 771.5182 | 3.67% | 0.03% | 5.93 | 2.15 | 0.36 | 2.12E-17 |
|  | PI (16:0/18:1) | -H | 11.8 | 835.5342 | 1.02% | 0.08% | 4.12 | 1.46 | 0.35 | 5.45E-15 |
|  | PI (16:0/22:6) | -H | 10.13 | 881.5186 | 2.07% | 0.01% | 4.65 | 1.75 | 0.38 | 5.05E-12 |
|  | PI (18:0/22:6) | -H | 11.5 | 909.5499 | 2.16% | 0.01% | 4.45 | 2.07 | 0.47 | 7.55E-10 |
|  | PI (18:1/18:1) | -H | 11.9 | 861.5499 | 1.00% | 0.16% | 5.12 | 1.91 | 0.37 | 1.26E-16 |
|  | PI (18:1/20:4) | -H | 10.57 | 883.5342 | 2.58% | 0.02% | 5.62 | 2.14 | 0.38 | 1.48E-18 |
|  | PS (18:0/18:2) | -H | 12.32 | 786.5291 | 11.33% | 0.02% | 5.22 | 1.78 | 0.34 | 1.08E-15 |
|  | PS (18:0/20:4) | -H | 12.09 | 810.5291 | 6.20% | 0.01% | 5.78 | 1.41 | 0.24 | 4.26E-20 |

Table S3. Forty-one lipid species differed amongst three lipid clusters (LClus).
