# Supplementary material for: Inflammatory responses relate to distinct bronchoalveolar lavage lipidome in community-acquired pneumonia patients: a pilot study
Source: Respir Res. 2019 May 2;20:82. doi: 10.1186/s12931-019-1028-8 (PMC6498485; doi:10.1186/s12931-019-1028-8)
Supplement: Supplementary file 2 — Tables S1. Summary of unique lipid species, by class, identified using LC-MS. Table S2. Thirty-three lipid species differentiated SCAP from controls. Table S3. Forty-one lipid species differed amongst three lipid clusters (LClus). Table S4. Correlation matrix of differential lipids of clusters and phagocyte percentages of BALF. (ZIP 123 kb) [file 12931_2019_1028_MOESM2_ESM.zip › Additional file 2. Table S4. Correlations of lipids and phagocytes in BALF.docx]

Table S4. Correlation matrix of differential lipids of clusters and phagocyte percentages of BALF.

| Phagocytes in BALF (%) | Lipid species | Spearman’s rank-order correlation | | Multivariate linear regression | |
| --- | --- | --- | --- | --- | --- |
|  |  | Coefficient | p-value | Adjusted coefficient | Adjusted p-value |
| Macrophages | TG (16:0/14:0/16:0) | 0.269 | 2.96E-03 |  |  |
|  | TG (16:0/16:0/16:1) | 0.277 | 2.21E-03 |  |  |
|  | TG (16:0/16:0/18:2) | 0.194 | 3.34E-02 |  |  |
|  | PC (16:0/20:4) | -0.250 | 5.89E-03 |  |  |
|  | SM (d34:1) | -0.436 | 6.61E-07 | -0.462 | 1.09E-7 |
|  | SM (d41:2) | -0.335 | 1.88E-04 |  |  |
|  | SM (d40:2) | -0.293 | 1.18E-03 |  |  |
|  | SM (d42:3) | -0.346 | 1.10E-04 |  |  |
|  | PE (18:0/20:4) | -0.248 | 6.47E-03 |  |  |
|  | PE (18:1p/20:4) | -0.306 | 6.66E-04 |  |  |
|  | FA (18:3) | -0.299 | 9.18E-04 |  |  |
| PMNs | FA (16:0) | -0.189 | 3.86E-02 |  |  |
|  | TG (16:0/14:0/16:0) | -0.260 | 4.07E-03 |  |  |
|  | TG (16:0/16:0/16:1) | -0.326 | 2.85E-04 |  |  |
|  | TG (16:0/16:0/18:2) | -0.296 | 1.02E-03 |  |  |
|  | PC (16:0/20:4) | 0.242 | 7.70E-03 |  |  |
|  | PI (18:0/22:6) | 0.180 | 4.92E-02 |  |  |
|  | SM (d34:1) | 0.326 | 2.74E-04 |  |  |
|  | SM (d41:2) | 0.291 | 1.27E-03 |  |  |
|  | SM (d40:2) | 0.299 | 9.02E-04 |  |  |
|  | SM (d42:3) | 0.262 | 3.89E-03 |  |  |
|  | PE (18:0/20:4) | 0.310 | 5.69E-04 |  |  |
|  | PE (18:1p/20:4) | 0.364 | 4.39E-05 | 0.541 | 1.79E-10 |
|  | PC (18:0/18:2) | 0.191 | 3.71E-02 |  |  |
|  | FA (18:3) | 0.400 | 5.96E-06 |  |  |
