# Supplementary figures and images for: Inflammatory responses relate to distinct bronchoalveolar lavage lipidome in community-acquired pneumonia patients: a pilot study
Source: Respir Res. 2019 May 2;20:82. doi: 10.1186/s12931-019-1028-8 (PMC6498485; doi:10.1186/s12931-019-1028-8)

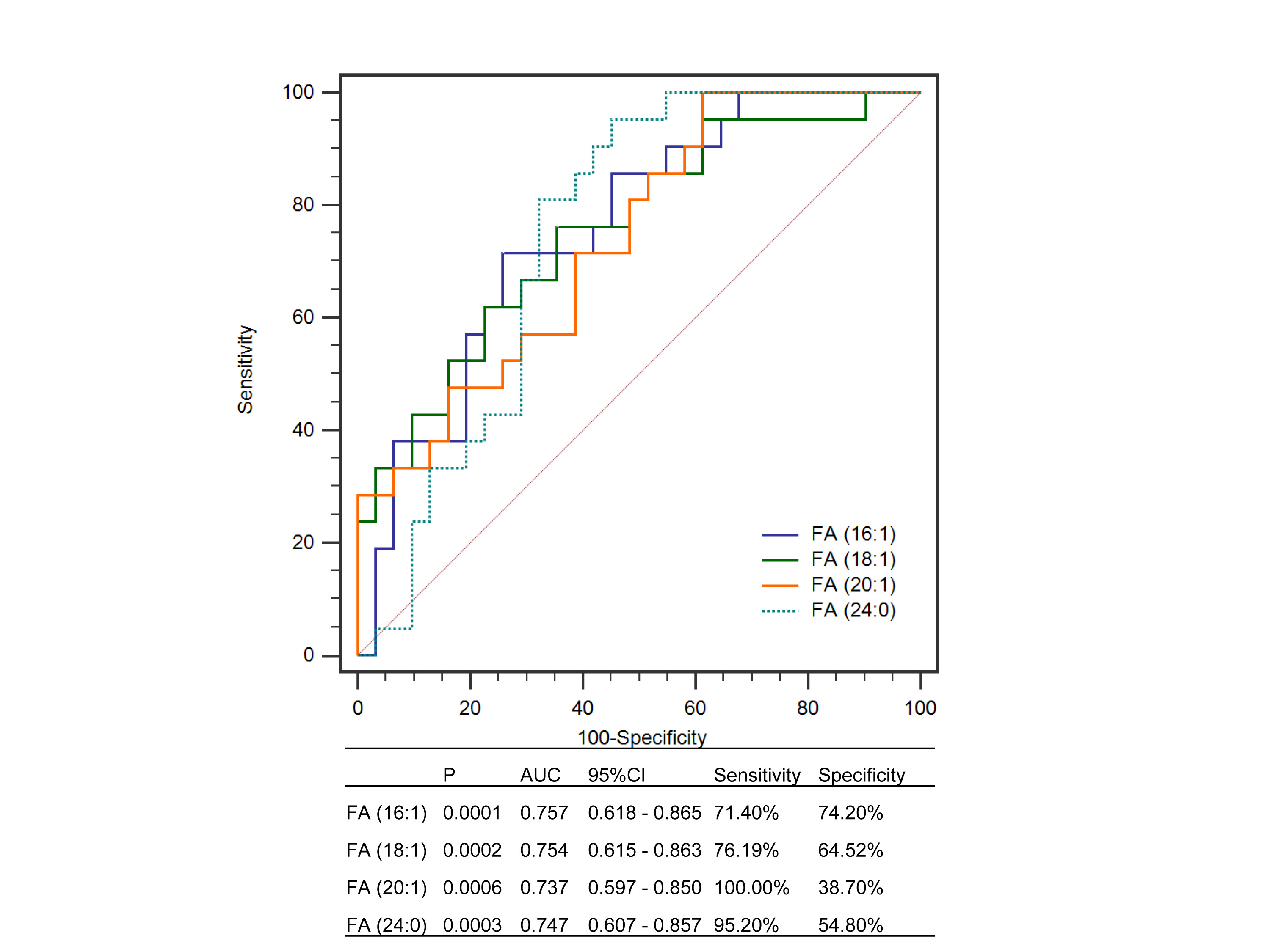

Supplement: Supplementary file 3 — Figure S1. Receiver operating characteristics (ROC) curves for FA (16:1), FA (18:1), FA (20:1), and FA (24:0) show the abilities to discriminate SCAP from NSCAP. Figure S2. Result of the elbow method to determine optimum number of clusters (k = 3). (ZIP 287 kb) [file 12931_2019_1028_MOESM3_ESM.zip › Additional file 3. FigureS1.jpg]

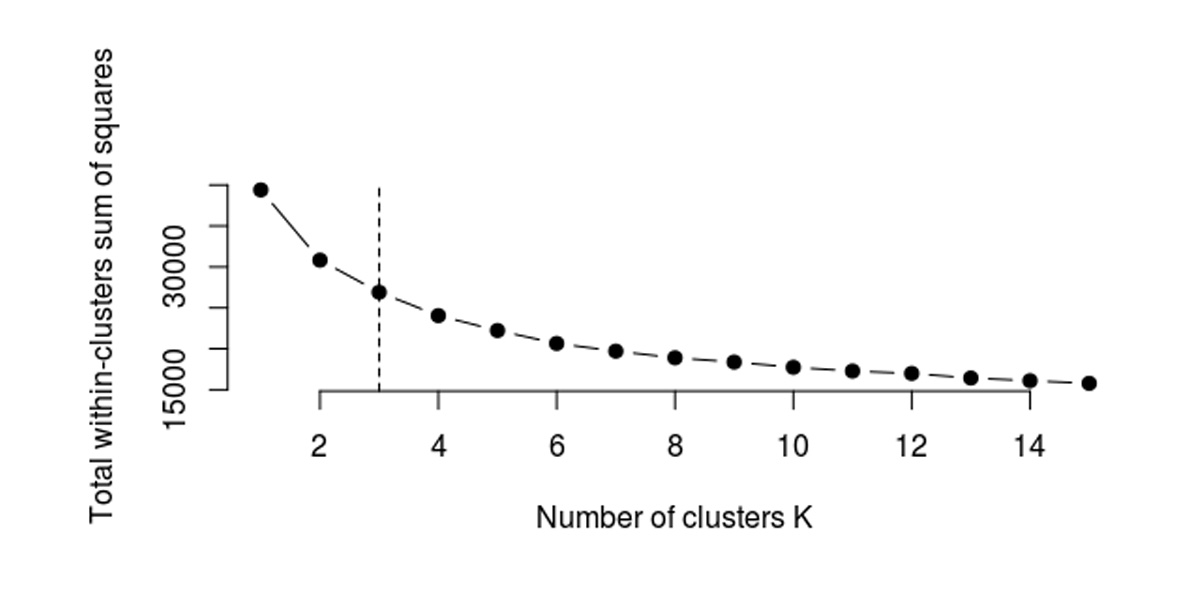

Supplement: Supplementary file 3 — Figure S1. Receiver operating characteristics (ROC) curves for FA (16:1), FA (18:1), FA (20:1), and FA (24:0) show the abilities to discriminate SCAP from NSCAP. Figure S2. Result of the elbow method to determine optimum number of clusters (k = 3). (ZIP 287 kb) [file 12931_2019_1028_MOESM3_ESM.zip › Additional file 3. FigureS2.jpg]
